# InsectGUILD: feeding guilds of lepidopteran and hymenopteran larvae consuming Northern Hemisphere woody plants

**DOI:** 10.1038/s41597-025-05229-9

**Published:** 2025-05-28

**Authors:** Andrea Cerdeira-Pérez, Lauri Laanisto, Giacomo Puglielli

**Affiliations:** 1 Departamento de Biología Vegetal y Ecología, Facultad de Biología, Universidad de Sevilla (US), Av. de la Reina Mercedes, 6, 41012 Sevilla, Spain; 2 Institute of Agricultural and Environmental Sciences, Estonian University of Life Sciences, Tartu, Estonia; 3 Department of Life Sciences, University of Trieste, Licio Giorgeri 10, 34127 Trieste, Italy

**Keywords:** Ecology, Forest ecology

## Abstract

The InsectGUILD dataset is an online data resource that compiles traits related to the feeding ecology and phenology for phytophagous butterflies, moths (Insecta: Lepidoptera) and sawflies (Insecta: Hymenoptera). We present traits on larval feeding guild (set of species that exploit the same type of resources in a similar way), larval host plant specificity (trophic specialisation) and voltinism (number of generations in a year) for a total of 4,818 species of the Lepidoptera in 84 families and 109 species of the Hymenoptera in 9 families. The lepidopteran and hymenopteran species subject of this collection have been recorded as feeders on dominant woody flora from the Northern Hemisphere. Features presented here are derived from online databases, field guides, and other literature resources. The InsectGUILD dataset resolves part of the data gap on large-scale insects’ feeding strategies in relation to host plants. By making these data freely available, we aim to provide an important data source for trait-based analyses exploring different research questions on interactions between insect herbivores and their associated host plant(s).

## Background & Summary

Nearly half of all known insect species feed on living plants^1^. Herbivorous (phytophagous) insects are one of the most abundant and diverse forms on Earth, and the consumption of plant material by insect herbivores is a dominant mover of energy and matter through terrestrial ecosystems, especially in forests^2^. Despite similar nutritional requirements, insects present a wide array of diets^3^. Phytophagous insects show a high variation in number of host plant species they feed on, as well as in their feeding niches and the way these niches are exploited^4^. The multiple ways in which insect consume plant tissues allows to structure herbivore communities across taxonomic boundaries, that is feeding “guilds” — defined here as groups of species using the same class of resources (e.g., leaves) in a similar way (e.g., exophytic behaviour)^5^. Different feeding guilds have detrimental effects on different plants’ parts, spanning reproductive, photosynthetic, and/or vascular/structural tissues, they influence population dynamics, as well as the evolution of the host plants^6–9^. However, information on insect feeding guilds is scant, often limited in geographical scope, and there is a lack of consistency across literature sources.

From the plants’ perspective, the consumption of plant material by phytophagous insects is detrimental to plant performance and fitness^10^, and plants have evolved a broad range of defense mechanisms to avoid or mitigate damages from insect herbivory^11^. However, the production and maintenance of defences against phytophagous insects’ costs energy for plants, thereby depending on habitat quality, including resource and non-resource stress levels. When resource and/or non-resource stress levels are high, plants must balance the allocation of resources needed to avoid/minimise the damage from phytophagous insects and to tolerate coexisting abiotic stressors. In other words, there must be an eco-evolutionary trade-off between biotic and abiotic stress tolerance^12^, but large-scale data to test this general hypothesis is missing. Recently, Puglielli *et al*.^13^ described the so-called Stress Tolerance Space (STS), a trade-off model defining the landscape of abiotic stress tolerance strategies towards four major abiotic stresses, namely shade, drought, cold, and waterlogging, for the dominant woody plants of the Northern Hemisphere. The STS provides us with a tool to explore the correlates of abiotic stress tolerance strategies in woody plants^14^. Winemiller *et al*.^15^ proposed a theoretical framework for defining a species niche by simultaneously considering a number of key ecological dimensions (i.e., habitat, life history, trophic, defense, and metabolic dimensions) in relation to species performance. Since the STS reflects species’ performance to given abiotic stress regimes in their natural habitat, Puglielli *et al*.^16^ proposed the integration of Winemiller’s *et al*.^15^ dimensions, including the defense dimension, with the STS model to contribute to disentangle how such key ecological dimensions co-determine the large-scale, realized abiotic stress tolerance strategies in woody plants.

Here, we present InsectGUILD, a dataset of feeding-related traits for 4,927 species of phytophagous insects known to feed on woody plants that define the STS. Feeding-related traits were derived from existing databases, relevant online resources, and published literature. The importance of InsectGUILD is two-fold: (i) it resolves part of the data gap on large-scale insects’ feeding strategies in relation to host plants; (ii) it provides a unique platform to explore the interaction between insects’ feeding strategies and plants abiotic stress tolerance strategies, since we specifically focused our search on the insect species that feed on the woody species that define the STS. For this synthesis effort, we focused on the plant-feeding life form (i.e., the larval stages) of Lepidoptera (butterflies and moths) and Hymenoptera (sawflies), which together constitute >30% of the known phytophagous insects^17^. The Lepidoptera represents the greatest diversification and radiation of any group of herbivores on the planet^18^, while some hymenopteran species were the sole feeders associated with certain wood plants under study.

## Methods

### Taxonomic and geographic coverage

We considered insects that have been recorded as feeders at their larval stages, and we were able to retrieve information for 81% (n = 645) of the woody species that define the STS (species constituting the majority of Northern Hemisphere woody biomass, from East Asia, Europe, and North America). For the woody species of the STS not covered in this dataset (19%), information on their phytophagous Lepidoptera and/or Hymenoptera was in fact lacking. The taxonomic status and accepted names of lepidopteran and hymenopteran species were validated and standardised using the fuzzy lookup tool from the Global Biodiversity Information Facility (https://www.gbif.org/tools/species-lookup, accessed: May 2024) and, in cases of missing information, we followed the Lepidoptera and Other Life Forms Database (https://ftp.funet.fi/index/Tree_of_life/insecta/, accessed: May, 2024). Synonyms were replaced with the accepted names. We retained accepted names with the highest confidence level provided by GBIF. In GBIF, names are standardised against multiple potential candidates, for each of which an overall confidence level is calculated based on several individual scores and ranked to select the best match. The highest-ranking candidate match is then presented to the user as the name with the highest confidence level. From an initial compilation of 5,187 insect species feeding on the woody plants that make up the STS dataset, 260 species are not part of the dataset presented here; the vast majority of these species are distributed in North America or East Asia. In most cases, information on the feeding mode was lacking, therefore limiting the possibility to define their feeding guild (see definitions below). Further, a significant part of the information for East Asian species was not available in English, which made interpretation and standardisation impossible. In cases where there was a risk of misinterpretation of the data, the species concerned was excluded from the dataset. This highlights potential priorities for future research.

### Data collection

Life-history traits related to the feeding ecology and phenology of the larval Lepidoptera and Hymenoptera were collected from 3 sources: (1) existing databases and relevant online resources including public collections and websites (representing the 11.5% of the total data sources), (2) published identification and field guides, books, and reports (35.5% of the total), and (3) papers from the primary literature (53.2% of the total). Sources for species-specific information are given in the database, and the complete list of data sources is presented in the dataset^19^.

Whenever possible, we retained the following information for each species: (1) the biomass pools or transport tissues exploited by the larvae (feeding niche). Online databases and websites (e.g., Plant Parasites of Europe, https://bladmineerders.nl; see the dataset^19^) were preferentially used rather than primarily literature because these sources are the result of a comprehensive effort to integrate and harmonise trait data across multiple and heterogenous data sources. Besides, data structure, terminology and definitions are presented following quality standards thus facilitating their use and interpretation; (2) the larval feeding mode (external or internal feeding). Online databases and websites were preferentially used rather than primarily literature; (3) host plant specialisation (diet breadth). Host plant specialisation data for lepidopteran species was primarily extracted from the Database of the World’s Lepidopteran Hostplants (HOSTS, https://www.nhm.ac.uk/our-science/data/hostplants/, accessed: May, 2024), while for hymenopteran species this information was mainly retrieved from the Lepidoptera and Other Life Forms Database. However, some authors^20^ noted a variety of errors within the HOSTS database, presumable related to larval misidentifications. When host plant records for a given species in the HOSTS database were clearly inconsistent with the other sources consulted, we retained data from sources among which there was consensus concerning host plant associations; and (4) voltinism (the number of generations per year). Online databases and collections were the primary source of voltinism information (see the dataset^19^). All larval host ranges and voltinism patterns for a given species were collected regardless of the geographical context. We therefore call for cautiousness when host range and voltinism are applied to a given study region, especially in the case of voltinism patterns, due to high variation of this trait across the latitudinal gradient. The collected traits and their definitions are provided in Table 1.

**Table 1:** List of species traits and descriptions within the InsectGUILD dataset.

Trait	Category	Explanation
**Feeding niche**	Cones/seeds	The dry, woody strobilus of a gymnosperm^39^
	Flowers	The sexual reproductive structure of angiosperms, typically consisting of an axis perianth part, androecium and gynoecium^40^
	Flower buds	Primary meristems inside protective covering containing embryonic and unexpanded flower parts^41^
	Fruits/seeds	The seed-bearing unit of angiosperms; it is the mature, ripened ovary and all of its associated protective covers, appendages, and supporting structures^39^.
	Leaves	The photosynthetic organs of a plant
	Leaf buds	Primary meristems inside protective covering containing unexpanded or undeveloped leaves with axillary growing points^41^
	Leaf gall	Growth or swelling within leaves caused by hypertrophy and/or hyperplasy of plant cells, induced by an organism, which provides nutrients and shelter for that organism^42^
	Shoots	Any young, tender, succulent, current-year, aerial outgrowth from a plant^43^
	Stem/trunk	This niche refers to the internal tissues of a stem or trunk; includes the phloem (inner bark; tissue concerned with translocation of foodstuffs) and xylem (the principal strengthening and water-conducting tissue)^43^
	Roots	Part of the underground axial system of a plant which does not bear leaves and tends to go downwards or laterally in the soil^40^
**Feeding mode**	External (exophytic)	Insect feeding on plant tissues that occurs externally on the plant
	Internal (endophytic)	Insect feeding on plant tissues that occurs within tissue of a living plant
**Hostplant specialisation**	Monophagous	Herbivore species restricted to feed on one or several plant species within a single genus^44^
	Oligophagous	Herbivore species that feeds two or more genera within one plant family^44^
	Polyphagous	Herbivore species that feeds as larva on plants from two or more families^44^
**Voltinism**	Univoltine	Species having one generation per year^45^
	Bivoltine	Species having two annual broods^45^
	Trivoltine	Species having three annual broods^45^
	Polyvoltine	Species having two or more generations per year (number of which may be unknown or unspecified)^45^
	2-year	A species that spends two years of its life cycle in the larval stage
	3-year	A species that spends three years of its life cycle in the larval stage

### Classification of larval feeding guilds

The larval Lepidoptera and Hymenoptera that make up the InsectGUILD dataset present diverse feeding ecologies, specialising in particular plant tissues via different modes of feeding. This information can be used to group species into functional groups, or feeding guilds, based on combining the information on the plant part consumed by the larvae, which can be defined as a particular plant organ, or plant tissue, with the information on how these are consumed, that is either internally or externally on the plant^21^. To try to minimize the uncertainty in defining the feeding guilds, we summarized the available information into the minimum possible number of guilds by broadly classifying them by the impact that the insect herbivory exerts on a given plant function, namely photosynthesis, water/sap transport and reproduction. We therefore defined the following guilds: “foliage consumption”, “consumption of transport tissues”, “damage to reproductive organs” (Table 2).

**Table 2:** Classification of feeding guilds (feeding niche x feeding mode) established for the lepidopteran and hymenopteran larvae included in the InsectGUILD dataset.

Feeding guild	Feeding niche	Feeding mode	Definition used in this paper
**Foliage consumption**			
Bud borer	Leaf buds	Internal	Species that bore into and feeds within leaf buds (i.e. undeveloped or embryonic shoot which occurs in the axil of a leaf or at the tip of a stem)
Bud chewer	Leaf buds	External	Species that feed externally on leaf buds
Leaf chewer	Leaves	External	Species that lives and feeds externally on the leaves or needles and remove leaf tissue that may or may not include leaf veins
Leaf galler	Leaf gall	Internal	Species that feed internally within leaves, where specific metabolic interactions result in differentiation of the plant tissue and subsequent abnormal growths referred to as galls
Leaf miner	Leaves	Internal	Organism that lives and feeds inside the blade of a leaf or needle, between the epidermal layers
Shoot feeder	Shoots	External	Species that feed externally on expanding leaves/needles of the shoots
**Damage to reproductive organs**			
Conospermatophage	Cones/seeds	Internal	Species that feeds internally on gymnosperms cones consuming seed-bearing structures and/or seeds
Flower borer	Flower buds; Flowers; seeds	Internal	Species that bore into and feeds internally within flower buds, flowers/catkins or developing seeds
Flower feeder	Flower buds; Flowers	External	Species that feeds externally on flowers, flower buds, and/or developing seeds
Fruit feeder	Fruits/seeds	Internal	Species that feeds internally within angiosperms fruits, consuming both seed-bearing tissues and seeds
**Consumption of transport tissues**			
Bark borer	Stem/trunk and/or branches	Internal	Species that bore into the woody portions of plants, feeding on the living tissue and disrupting transport
Root borer	Roots	Internal	Species that bore into and feeds within internal tissues of the roots
Root feeder	Roots	External	Species that feed externally on the roots
Shoot borer	Shoots	Internal	Species that lives and feeds within expanding shoots of host plants. Shoot: the current new growth of a branch tip

### Quantitative classification of feeding strategies and larval feeding guilds

Categorizing herbivorous larvae into discrete feeding guilds is a common way for grouping species into a reduced number of functional categories reflecting shared plant niches and feeding behaviours. On the other hand, the degree of niche specialisation and the feeding modes used by the larvae can differ substantially from one species to another. In cases where species depend on a single plant organ, or tissue, the classification is straightforward, since they are assigned to a single feeding guild. When larvae exploit different plant niches they are assigned to different feeding guilds, and there are multiple feeding guild combinations across species in the dataset. To facilitate the comprehension of the data, and to promote future quantitative analyses, we provide a quantitative classification and examination of the feeding functional groups contained within the InsectGUILD dataset.

#### Main axes of variation in feeding strategies across species

**Fig. 1:**
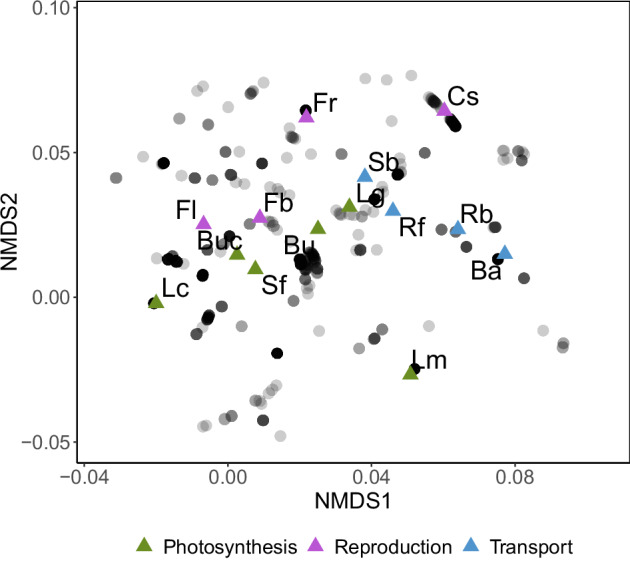
The feeding strategy space for insects’ larvae in the InsectGUILD dataset. Non-metric multidimensional scaling (NMDS) based on Gower dissimilarities (stress = 0.033). Black points show individual species (n = 4,927). Lighter (grey) points indicate a lower local density of species in the feeding strategy space. Discrete feeding guilds mapped across the feeding space are represented by triangles, which are coloured according to the fitness impacts of phytophagous larvae on the plant host(s). Ba: bark borer; Bu: bud borer; Buc: bud chewer; Cs: conospermatophage; Fb: flower borer; Fl: flower feeder; Fr: fruit feeder; Lc: leaf chewer; Lg: leaf galler; Lm: leaf miner; Rb: root borer; Rf: root feeder; Sb: shoot borer; Sf: shoot feeder.

Trait information was re-coded by indicating the partial-to-full membership of each species to a given feeding guild category (going from 0% to 100%). For example, a species that belongs to a single feeding guild is assigned 1 for that feeding guild and 0 for the other guilds. If a species belongs to two feeding guilds, then the species is assigned 0.5 for each feeding guild. The same logic was applied for species belonging to > 2 feeding guilds. We used these re-coded data to define the main axes of variation in feeding guilds across species by performing a non-metric multidimensional scaling (hereafter NMDS) based on Gower distances using the metaMDS function from the *vegan* R package^22^. With this method, similar objects are plotted close to one another in the ordination space, while distant objects are characterized by a greater dissimilarity^23^. The resulting ordination from NMDS showed distinct positions among insects’ feeding behaviours that defined the space of species’ feeding strategies (Fig. 1). The first NMDS axis (NMDS1) mainly separated species along a spectrum from external (exophytic) to internal (endophytic) feeders, with leaf chewers (external leaf-feeding larvae) and bark borers (endophytic larvae exploiting transport tissues) at the highest and at the lowest end of the spectrum, respectively. The second NMDS axis (NMDS2) mainly sorted species according to their feeding niche, with larvae exploiting tree reproductive structures (conospermatophages and fruit feeders) and leaf miners (larvae feeding within leaves) at the highest and the lowest end of the spectrum, respectively.

Phytophagous insects have evolved a range of morphological, physiological, and behavioural characteristics to exploit a variety of plant niches available in terrestrial ecosystems^17^. While the NMDS identifies the main axes of specialization in insects feeding strategies, we also wanted to objectively identify and describe the end-point strategies of the defined the feeding strategy space. To this aim, we further characterised the configuration of the feeding strategy space in terms of Pareto-like optimality principles^24^ by conducting an archetypal analysis using the *archetypes* R package^25^. This method aims at identifying irregular polygons defined by k vertices, or ‘archetypes’, defined as well-separated observations representing a phenotype more tightly associated to a specific functional objective (i.e., specialised feeding behaviour). The functional identity of every data point is subsequently characterised as a convex combination of these primary functions^24,26,27^ and the edges of these polygons can be interpreted in terms of trade-offs (or Pareto front^17^) limiting potential combinations of target features. Since we did not have any a priori expectation on the best fitting polygon, we iteratively assessed it using a number of vertices ranging from k = 2 to 7 using 100 replications at each iteration^25^.

**Fig. 2:**
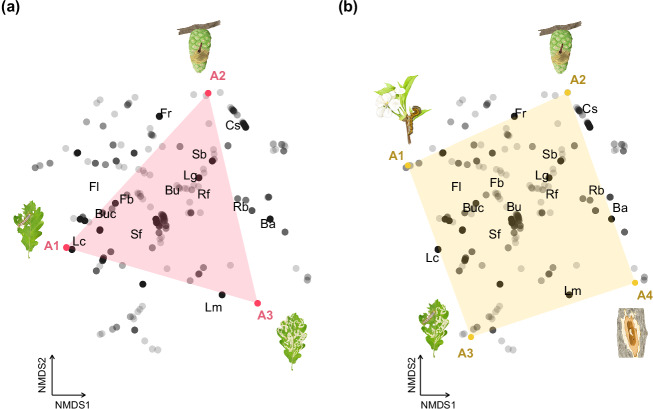
Best fitting polygons describing the feeding strategy space. Colour polygons represent the convex hull of the archetypes identified from the (**a**) three- (rss = 0.003) and (**b**) four- (rss = 0.006) archetypes models (n = 4,927). The position of archetypes, labelled as A1, A2, A3, and A4, is shown by colour points. Illustrations represent feeding behaviours close to archetypal positions.

The insect feeding strategy space presented here can be well described by either a triangle or a trapezoid (Fig. 2). In the first model (rss = 0.003; Fig. 2a), the first archetype (A1) of the feeding strategy space was maximally enriched with leaf chewers, while archetype 3 (A3) described endophagous larvae exploiting leaves (leaf miners). The enclosed environment of the leaf miners provides protection against external abiotic factors and access to more nutritionally rich tissue layers within leaves, but also imposes physical restrictions thereby limiting insects’ body size, number of generations per year (voltinism), or the capability to colonise alternative hosts and habitats^4^. Moreover, individuals are fully exposed to the plants’ chemical defenses. The intimate physical and functional relationship with the plant host requires specific and fine adaptations and a relatively high degree of host specialisation compared to external feeders^4,28^. The second archetype (A2) was maximally enriched with endophytic larvae consuming the fruiting structures (seeds and/or seed-bearing tissues) of angiosperms (fruit feeders) or gymnosperms (conospermatophages). Fruits and cones are well-defended structures that differ qualitatively from vegetative parts of the tree, presenting very high nutrient values per unit of volume. Besides physical and chemical barriers, fruiting structures are available on the plant for a brief period, and seed production can be highly irregular in both space and time^29,30^. Insects feeding on tree reproductive structures thus represent a strong trophic specialisation and as a result is limited to a highly specific entomofauna^29^. Besides, this strategy is nuanced by the various ways in which these insects exploit very different reproductive organs (i.e., cones, seeds, flowers)^9,31^.

The first archetype (A1) identified in the second model (rss = 0.001; Fig. 2b) was maximally enriched with larvae evolved to feed on flowers, flower buds, and leaf buds. The shoots are actively growing tissues with a high nutritive value, and generally present comparatively lower amounts of chemical and physical defenses than older tissues, being therefore more accessible to herbivore attack^32^. As observed in the previous model, the second archetype (A2) described larvae feeding on reproductive structures, while the third archetype (A3) was positioned at an intermediate location between leaf chewers and leaf miners. Some lepidopteran species within the dataset, mainly represented by members of the Gracillariidae family, are leaf miners at early preadult instar stages and later instars move to external leaf feeding. This apparently “transitional” trait may reflect a strategy to avoid the negative consequences of internal feeding^28,33^. Finally, the fourth archetype (A4) describes larvae that bore into the stem or trunk and feed on the cambium, xylem and/or phloem tissues. The hallmark of this archetype is the unbalanced nutrient composition of the insects’ feeding niche^34,35^. The capability of some insects to adopt certain nutritional behaviours rely not only on the insects’ feeding-related traits but also on the alliance of insects and microorganisms presenting different biosynthetic or degradative capabilities^3^. Indeed, the recent work by Cornwallis *et al*.^36^ showed that over 90% of insect species feeding on phloem and xylem present obligate symbiosis with microorganisms (including bacteria, fungi and protist symbionts), whereas this association only represent less than 10% of insects that have more nutritious and/or balanced diets (e.g., leaves, flowers, seeds and/or root tips). This insect-microbiome dependence and collaboration facilitated the transition of phytophagous larvae to previously inaccessible, specialised, and nutrient imbalanced feeding niches. Table 3 summarises the feeding guilds associated with the vertices (archetype positions) of the polytopes identified in each archetypes model.

**Table 3:** Insect feeding guilds close to archetypal positions (A1–A4) in the three- (k = 3) and four-archetypes (k = 4) models.

	A1	A2	A3	A4
k = 3	Leaf chewer	Conospermatophage- Fruit feeder	Leaf miner	—
k = 4	Flower feeder- Shoot feeder	Conospermatophage	Leaf chewer- Leaf miner	Bark borer

We argue that the triangular polytope better captures the principal functional trade-offs of the feeding strategy space depicted by the NMDS than the trapezoidal polytope. The latter model appears in fact to overfit the feeding strategy space (all data points are included in the polygon), suggesting that it is a more descriptive-like model of the data. The quantitative framework presented here allows us to summarize and describe the main axes of variation of insect feeding strategies, which are constrained by trade-offs among three end-point strategies that constraint such strategies within a triangular polygon.

#### Assigning species to feeding guilds in a continuous fashion

To complement the previous analyses, we further examined the distribution of feeding guilds in the feeding strategy space through a fuzzy clustering analysis implemented in the R package *fclust*^37^. Unlike ‘hard’ clustering, which assigns each observation exclusively to one cluster, the fuzzy *k*-means clustering method estimates the likelihood of each observation belonging to a certain cluster^38^. Hence, each species is assigned to each cluster with an associated membership value continuously varying between 0 (no class membership) and 1 (highest degree of cluster membership). The number of clusters was assessed within the range of 2 to 7, and the best cluster structure was evaluated through the partition coefficient (PC), partition entropy (PE), fuzzy silhouette (SIL.F) and Xie and Beni (XB) validity indexes available in the package.

**Fig. 3:**
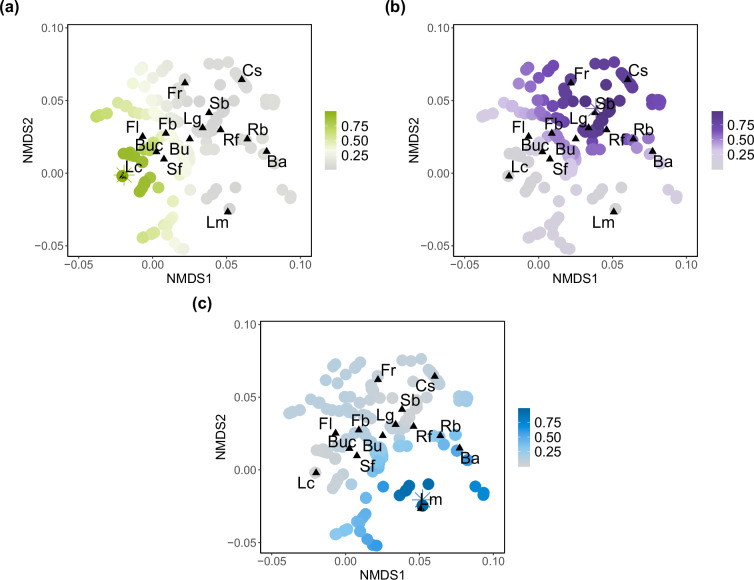
Fuzzy k-means (fkm) clustering of feeding strategies based on NMDS ordination (n = 4,927 species). Stars show the position of the prototype representing each of the three clusters. Colour intensity is related to the probability of a species belonging to the cluster it has been classified into. Membership degrees range in the interval [0-1].

The fuzzy k-means (fkm) clustering algorithm identified 3 groups of insects presenting similar feeding patterns (Fig. 3). The largest cluster (cluster 1) included 3,581 species (72% of the total) that were mostly external feeders: leaf chewers, bud chewers, shoot feeders, and flower feeders (Fig. 3a). Cluster 2 included 436 species (9%) and was characterised by internal feeders: leaf gallers, shoot borers, and larvae exploiting reproductive structures of angiosperms (fruit feeders) and gymnosperms (conospermatophages). On the contrary, larvae exploiting mature leaves, either externally or internally presented the lowest membership degree for cluster 2 (Fig. 3b). Finally, the third cluster included 910 species (18%) and was dominated by leaf miners. Bark borers were also assigned to this third cluster (Fig. 3c).

The three-clusters classification supports the triangular organisation drawn by archetype analysis. Thus, cluster membership degree represents a ternary scoring system expressing the inherent trade-offs among the three end-point strategies that define the polygon in Fig. 2a, allowing to assign species to feeding strategies within the three-vertex polytope in a continuous fashion.

## Data Records

The InsectGUILD dataset^19^ is available at figshare. The complete dataset consists of 7 files; (1) “Feeding_ecology_traits.csv” data file, which contains the raw traits information table; (2) “Feeding_axes.csv” data file, which contains the coordinates obtained from NMDS analysis (see above) for each insect species. This allows to quantitatively and continuously sort species based on their feeding guild(s); (3) “Guild_membership.csv” data file, which presents, for each insect species, the maximal cluster membership degree values derived from the fuzzy clustering analysis; (4) “Insect_host_associations” data file, which presents a list of the insect species that make up the dataset by plant host(s) (n = 645); (5) “Recoded_trait_data.csv” data file, which contains re-coded raw trait information used to define the main axes of variation in feeding guilds through a non-metric multidimensional scaling (NMDS); (6) “References.docx” text file, which contains all the references included as numbers in the raw trait information table; and (7) “Analysis_code.R” file, that contains the code to reproduce the analyses.

### Data summary

Our dataset includes trait information for a total of 4,818 lepidopteran species in 84 families and 109 hymenopteran species in 9 families. The species coverage of trait information is complete for the feeding niche, mode of feeding and host plant specialisation, whereas a low level of completeness was achieved for voltinism (54%, n = 2666) (Table 4). The notation NA (not available) was used for missing data in the dataset.

**Table 4:** The number of species represented within each family and data completeness in InsectGUILD.

Order	Family	# Species	Data completeness (%)		
			Feeding_guild	Host_plant specialisation	Voltinism
Lepidoptera	Adelidae	1	100	100	100
	Agonoxenidae	1	100	100	100
	Anthelidae	5	100	100	0
	Apatelodidae	2	100	100	50
	Arctiidae	26	100	100	59
	Autostichidae	1	100	100	0
	Batracheridae	4	100	100	50
	Blastobasidae	9	100	100	33
	Bombycidae	1	100	100	0
	Bucculatricidae	20	100	100	45
	Carposinidae	6	100	100	50
	Chimabachidae	3	100	100	0
	Choreutidae	5	100	100	20
	Coleophoridae	82	100	100	95
	Copromorphidae	1	100	100	0
	Cosiidae	1	100	100	100
	Cosmopterigidae	18	100	100	55
	Cossidae	12	100	100	25
	Crambidae	32	100	100	35
	Dalceridae	2	100	100	50
	Depressariidae	50	100	100	42
	Drepanidae	37	100	100	62
	Elachistidae	21	100	100	67
	Endromidae	1	100	100	0
	Epermeniidae	2	100	100	0
	Epicopeiidae	1	100	100	100
	Erebidae	297	100	100	53
	Eriocraniidae	10	100	100	90
	Eupterotidae	2	100	100	0
	Euteliidae	7	100	100	29
	Galacticidae	1	100	100	100
	Gelechiidae	240	100	100	41
	Geometridae	660	100	100	56
	Glechiidae	2	100	100	50
	Glyphipterigidae	1	100	100	0
	Gracillariidae	370	100	100	33
	Heliozelidae	14	100	100	36
	Hepialidae	9	100	100	33
	Hesperiidae	28	100	100	82
	Hyblaeidae	1	100	100	100
	Incurvariidae	6	100	100	17
	Lacturidae	1	100	100	0
	Lasiocampidae	68	100	100	47
	Lecithoceridae	6	100	100	0
	Limacodidae	48	100	100	31
	Lycaenidae	142	100	100	71
	Lymantriidae	1	100	100	0
	Lyonetiidae	16	100	100	25
	Megalopygidae	11	100	100	36
	Metarbelidae	1	100	100	100
	Mimallonidae	2	100	100	50
	Momphidae	4	100	100	75
	Nepticulidae	152	100	100	68
	Noctuidae	503	100	100	61
	Nolidae	39	100	100	49
	Notodontidae	144	100	100	58
	Nymphalidae	100	100	100	84
	Oecophoridae	9	100	100	33
	Opostegidae	3	100	100	33
	Pantheidae	1	100	100	100
	Papilionidae	69	100	100	58
	Pieridae	24	100	100	75
	Prodoxidae	7	100	100	57
	Psychidae	40	100	100	45
	Pterophoridae	8	100	100	75
	Pyralidae	177	100	100	55
	Riodinidae	3	100	100	67
	Roeslerstammiidae	1	100	100	100
	Saturniidae	247	100	100	43
	Schreckensteiniidae	2	100	100	0
	Scythrididae	6	100	100	17
	Sesiidae	74	100	100	74
	Sphingidae	83	100	100	75
	Stathmopodidae	1	100	100	0
	Thyrididae	2	100	100	50
	Tineidae	3	100	100	67
	Tischeriidae	15	100	100	27
	Tortricidae	709	100	100	41
	Urodidae	1	100	100	100
	Xyloryctidae	3	100	100	0
	Yponomeutidae	82	100	100	78
	Ypsolophidae	10	100	100	30
	Zygaenidae	8	100	100	57
Hymenoptera	Argidae	13	100	100	38
	Cephidae	3	100	100	67
	Cimbicidae	19	100	100	58
	Diprionidae	17	100	100	100
	Pamphiliidae	29	100	100	19
	Siricidae	8	100	100	100
	Tenthredinidae	11	100	100	75
	Xiphydriidae	5	100	100	0
	Xyelidae	4	100	100	0

## Technical Validation

The primary source of information for trait records included in the dataset derives from institutional databases, public collections, and websites, as well as from published material in peer-reviewed scientific journals and books; thus, we have confidence in their accuracy. Moreover, we have checked the initial version of the dataset for possible errors and redundancies, removing duplicated entries and synonymised names. Data will be corrected and updated directly on figshare if any errors or updates are reported by users to the corresponding author.

## Supplementary information

References

readme_InsectGUILD

Analysis_code.R

## Data Availability

The link to the InsectGUILD dataset^19^ and the R code used to reproduce the analyses are available at figshare under the terms of a Creative Commons Attribution 4.0 International waiver. The CC-BY-4.0 waiver facilitates the discovery, re-use and citation of the dataset. Future updates of the dataset will be made on figshare.
