# Supplementary material for: InsectGUILD: feeding guilds of lepidopteran and hymenopteran larvae consuming Northern Hemisphere woody plants
Source: Sci Data. 2025 May 28;12:887. doi: 10.1038/s41597-025-05229-9 (PMC12119911; doi:10.1038/s41597-025-05229-9)
Supplement: Supplementary file 1 — References [file 41597_2025_5229_MOESM1_ESM.docx]

**InsectGUILD: feeding guilds of lepidopteran and hymenopteran larvae consuming Northern Hemisphere woody plants**

Reference sources used for data compilation. Numbers are used within the dataset to identify individual sources of data for each of the insect species included in the InsectGUILD dataset.

1. Aarvik, L. & Christiansen, C. Xestia gelida (Sparre Schneider, 1883)(Lepidoptera, Noctuidae) rediscovered in Norway. *Nor. J. of Entomol.* **58**, 1–6 (2011).
2. African Moths <https://africanmoths.com/>.
3. Agriculture Department of Illinois. *Transactions of the Department of Agriculture of the State of Illinois with reports from county and district agricultural organizations for the year*. vol. 38. (1871). <https://www.biodiversitylibrary.org/item/21781> (1871)
4. Agriculture Department of Illinois. *Insects of Eastern forests.* vol. 1426 <https://www.biodiversitylibrary.org/item/131979> (U.S. Forest Service, 1985).
5. Ahmed, N. Study of insect pests of poplar in Pakistan. *The Pakistan Journal of Forestry* **67**, 23–29 (2017).
6. Alford, D. V. *Pests of fruit crops: A color handbook. Plant protection handbooks.* (2007).
7. Alston, D. G. & Reding, M. E. “Western Tentiform Leafminer Phyllonorycter elmaella” <https://digitalcommons.usu.edu/extension_curall/1923> (2011).
8. Aizpurúa C. Identificación de cinco orugas minadoras, huéspedes de pinos. *Bol. San. Veg. Plagas,* **20***,* 833-–845 (1994).
9. Ameen, M. U. & Sultana, P. Biology of the bag-worm moth Eumeta crameri Westwood (Lepidoptera: Psychidae) from Dacca, Bangladesh. *J. Nat. Hist.* **11,** 17–24 (1977).
10. An Identification Guide of Japanese Moths <http://www.jpmoth.org> (2024).
11. Antram, C. B. *Butterflies of India.* (Mittal Publications, 1924).
12. Arguedas-Gamboa, M. Plagas y enfermedades forestales en Costa Rica. *Revista Forestal Mesoamericana Kurú*, **4,** 11–12 (2007).
13. Área de Conservación de Guanacaste. Páginas de Especies-Insectos <https://www.acguanacaste.ac.cr/paginas-de-especies/insectos/> (2024).
14. Arita, Y. Descriptions of the larva and pupa of Similipepsis takizawai Arita & Spatenka (Lepidoptera. Sesiidae). *Nota Lepid.*, **13,** 192–197 (1990).
15. Arita, Y. The Clearwing Moths of Japan (Lepidoptera: Sesiidae). *Holarct. Lepid.* **1,** 69–81 (1994).
16. Arita, Y., Niimi, S., & Nakano, H. Descriptions of the larva and pupa of a clearwing moth Synanthedon multitarsus Spatenka & Arita (Sesiidae). *Lepidoptera Science* ***43*,** 239–244 (1993).
17. Arnett Jr, R. H. *American insects: a handbook of the insects of America north of Mexico.* (Crc Press, 2000).
18. Arora, G. S. Taxonomic studies of some of the Indian non-mulberry silk moths (Lepidoptera: Saturniidae). *Memoirs Zool. Surv. India* **16,** 11pls (1979).
19. Arun, P. R., & Mahajan, M. v. Ecological costs and benefits of Teak Defoliator (Hyblaea puera Cramer) outbreaks in a mangrove ecosystem. *Mar. Sci.* **2,** 48–51 (2012).
20. Ashmead, W. M. H. Injurious and beneficial insects found on the orange trees of Florida. *Can. Entomol.* **11,** 159–160 (1879).
21. Atherton, J. N., McKenna, S. A., & Wheatley, A. Rapid Biodiversity Assessment of the Vava’u Archipelago, Kingdom of Tonga (2014).
22. Atlas of Living Australia  [*http://www.ala.org.au*](http://www.ala.org.au)*.* (2024).
23. Ayre, G. L., & Lamb, R. J. Life histories, flight patterns, and relative abundance of nine cutworms (Lepidoptera: Noctuidae) in Manitoba. *Can. Entomol.* **122,** 1059–1070 (1990).
24. Baixeras, J., Domínguez, M., & Martínez, S. El género Rhyacionia Hübner [1825] en la Península Ibérica (Lepidóptera, Tortricidae). Boletín de Sanidad Vegetal. *Plagas*, **22,** 711–730 (1996).
25. Baker, W. L. *Eastern forest insects*. vol. 1166 (US Forest Service, 1972).
26. Baker, W. L. (Whiteford L. *Eastern forest insects: Vol. no.1175*. U.S. Dept. of Agriculture, Forest Service <https://www.biodiversitylibrary.org/item/132738> (1972).
27. Bąkowski, M. Redescription of female of Paranthrene insolita polonica Schnaider 1939 (Lepidoptera: Sesiidae). *Genus* **20,** 359–366 (2009).
28. Baranchikov, Y. N., Maitson, W. J., Hain, F. P. & Payne, T. L. *Forest insect guilds: patterns of interaction with host trees*. Forest Service- General Technical Report NE-153 ( US Department of Agriculture, 1989).
29. Baranowski, A. K., Alm, S. R. & Preisser, E. L. Datana drexelii (Lepidoptera: Notododontidae) occurrence and larval survival on highbush blueberry cultivars. *J. Econ. Entomol.* **113,** 1568–1571 (2020).
30. Barnes, W. & McDunnough, J. On the early stages of some western Catocala species. *Psyche: A Journal of Entomology*, **20,** 188–202 (1913).
31. Barras, S. J. & Norris, D. M. Bionomics of Eucosma monitorana (Lepidoptera: Tortricidae) attacking red pine cones in Wisconsin. *Ann. Entomol. Soc. Am.* **62**, 1284–1290 (1969).
32. Barrera, G., Barrera, G., Gómez, J., Cuartas, P., León, G. & Villamizar, L. Caracterización morfológica, biológica y genética de un aislamiento Colombiano de granulovirus de Erinnyis ello (L.)(Lepidoptera: Sphingidae). *Revista Colombiana de Biotecnología* **16**, 129–140 (2014).
33. Barrios-Díaz, B., Sánchez-García, L., Equihua–Martínez, A., Vázquez-Huerta, G. & Barrios-Díaz, J. M. Identificación de lepidópteros asociados al follaje de encino (Quercus spp.), en el municipio de Tetela de Ocampo, Puebla. *Entomol. Mex.* **4**, 537–543 (2017).
34. Basso, A., Simonato, M., Cerretti, P., Paolucci, P. & Battisti, A. A review of the “summer” Thaumetopoea spp.(Lepidoptera: Notodontidae, Thaumetopoeinae) associated with Cedrus and Pinus. *Turkish Journal of Forestry* **17**, 31–39 (2016).
35. Beaver, E. P. Revision of the genus Metura (Lepidoptera: Psychidae) with description of two new species. *Zootaxa* **4861**, 188–210 (2020).
36. Bedding, R. A., Akhurst, R. J. & Kaya, H. K. *Nematodes and the biological control of insect pests*. (Csiro Publishing, 1993).
37. Bentancourt, C. M., Scatoni, I. B., Gonzalez, A. & Franco, J. Biology of Bonagota cranaodes (Meyrick)(Lepidoptera: Tortricidae) on seven natural foods. *Neotrop. Entomol.* **33**, 299–306 (2004).
38. Bentancourt, C. M., Scatoni, I. B., Gonzalez, A. & Franco, J. Biology of Bonagota cranaodes (Meyrick)(Lepidoptera: Tortricidae) on seven natural foods. *Neotrop. Entomol.* **33**, 299–306 (2004).
39. Bernardo, U. *et al.* Characterization, distribution, biology and impact on Italian walnut orchards of the invasive North-American leafminer Coptodisca lucifluella (Lepidoptera: Heliozelidae). *Bull. Entomol. Res.* **105**, 210–224 (2015).
40. Berndt, L. A., Berry, J. A. & Brockerhoff, E. G. Parasitoids and predators of the endemic defoliator Pseudocoremia suavis (Butler)(Lepidoptera: Geometridae: Ennominae). *N. Z. Entomol.* **29**, 89–98 (2006).
41. Bhatt, J. *Butterflies of Pithoragarh (A Field Guide)*. (2013).
42. Bidzilya, O. A review of the genus Athrips (Lepidoptera, Gelechiidae) in the Palaearctic region. *Dtsch.Entomol. Z.* **52**, 3–72 (2005).
43. Bigger, M. A geographical distribution list of insects and mites associated with cocoa, derived from literature published before 2008 [*http://www.ipmnetwork.net/commodity/cocoa_insects.pdf*](http://www.ipmnetwork.net/commodity/cocoa_insects.pdf) (2008).
44. Bland, K. P., Hancock, E. F. & Razowski, J. *Tortricidae, part 1: Tortricinae & Chlidanotinae*. vol. 5 (Brill, 2015).
45. Bland, K. P., Razowski, J. & Hancock, E. F. *Tortricidae, part 2: Olethreutinae*. vol. 5 (Brill, 2015).
46. Böcher, J., Kristensen, N., Pape, T. & Vilhelmsen, L. *The Greenland Entomofauna. An Identification Manual of Insects, Arachnids and their Allies* <https://doi.org/10.1163/9789004261051> (2015)
47. Bong-Kyu, B., Shan-chun, Y. & Cheng-de, L. Notes on genus Eurydoxa Filipjev (Lepidoptera: Tortricidae) in China. *J. For. Res.* **14**, 210–212 (2003).
48. Bong-Kyu, B., Shan-chun, Y. & Cheng-de, L. Revision of tribe archipini (Tortricidae: Tortricinae) in northeast China. *J. Fore. Res.* **14**, 93–102 (2003).
49. Bourquin, F. Notas sobre la metamorfosis de Pseuderotis cannescens Clarke, 1956 (Lepidop. Oecophoridae). *Rev. Soc. Entomol. Argent.* **19**, (1956).
50. Braby, M. F. *Butterflies of Australia: their identification, biology and distribution*. (CSIRO publishing, 2000).
51. Braby, M. F. *The complete field guide to butterflies of Australia*. (Csiro Publishing, 2004).
52. Braby, M. *et al.* *Atlas of butterflies and diurnal moths in the monsoon tropics of Northern Australia*. (ANU Press, 2018).
53. Braun, A. F. Nepticulidae of North America. *Trans. Am. Entomol. Soc. (1890-)* **43**, 155–209 (1917).
54. Braun, A. F. Microlepidoptera of northern Utah. *Trans. Am. Entomol. Soc. (1890-)* **51**, 183–226 (1925).
55. Braun, A. F. Notes and new species of Microlepidoptera from the Mineral Springs region of Adams County, Ohio. *Trans. Am. Entomol. Soc. (1890-)* **56**, 1–17 (1930).
56. Braun, A. F. The genus Bucculatrix in America North of Mexico (Microlepidoptera). *Memoirs of the American Entmological Society Philadelphia* **18**, 1–208 (1963).
57. Brock, J. P., Brock, J. P. & Kaufman, K. *Kaufman field guide to butterflies of North America* (Houghton Mifflin Harcourt, 2003).
58. Brou, Jr. Acronicta hammelis Guenée, 1852 (Lepidoptera: Noctuidae). *Louisiana. South. Lepid. News* 38–16 (2016).
59. Brou, V. A. Two new species of Baileya Grote (Nolidae: Risobinae) from the southeastern United States. *Journal-Lepidopterists Society* **58**, 94–99 (2005).
60. Brown, J. W. A review of host plants for the tortricid tribe Grapholitini, with a synopsis of host utilization by genus (Lepidoptera: Tortricidae). *Insecta Mundi* **0944,** 1–75 (2022).
61. Brown, J. W., Miller, S. E., Rosati, M. E., Copeland, R. S., Aarvik, L. & Luke, Q. Host records for fruit-feeding Afrotropical Tortricidae (Lepidoptera). *Afr. Entomol.* **22,** 343–376 (2014).
62. Brown, J. W. & Passoa, S. Larval foodplants of Euliini (Lepidoptera: Tortricidae): from Abies to Vitis. *Pan-Pacific Entomologist* **74,** 1–11(1998).
63. Brown, J. W. & Powell, J. A. *Systematics of the Chrysoxena group of genera (Lepidoptera: Tortricidae: Euliini)*. vol. 111. (Univ of California Press, 1992).
64. Brown, J. W. & Powell, J. A. *Systematics of Anopina Obraztsov (Lepidoptera Tortricidae: Euliini)*. vol. 120. (Univ of California Press, 2000).
65. Brown, L. N. & Mizell, R. F. The clearwing borers of Florida (Lepidoptera: Sesiidae). *Trop. Lepid. Res.* **4,** 1-21 (1993).
66. Bruun, H. H. Prospects for biocontrol of invasive Rosa rugosa. *Biocontrol* **51**, 141–181 (2006).
67. Bryant, P. J. Natural History of Orange County, California and nearby places <https://nathistoc.bio.uci.edu/index.htm>.
68. Buckett, J. S. Revision of nearctic genus Philtraea Hulst, with notes on biology and the descriptions of new species (Geometridae). *J. Res. Lepid.* (1970).
69. Burdick, D. J. & Powell, J. A. Studies on the early stages of two California moths which feed in the staminate cones of digger pine (Lepidoptera: Gelechiidae). *Can. Entomol.* **92**, 310–320 (1960).
70. Burns, R. M. *Silvics of north America* (US Department of Agriculture, Forest Service, 1990).
71. Busck, A. A new Moth injurious to Loquat in Argentina. *Anales de la Sociedad Científica Argentina* **5,** 361-365 (1939).
72. Bustillo Pardey, A. E. Estudios del gusano rojo peludo, Lichnoptera gulo Herrich-Schaeffer (Lepidoptera: Noctuidae), plaga del pino y ciprés, 1: biología y ecología (2018).
73. Butterfly Conservation South Australia Inc (BCSA). South Australian Butterflies & Moths. Data Sheet "Dingy or Dainty Swallowtail Papilio (Eleppone) anactus (W.S. Macleay) [*https://sabutterflies.org.au/papi/anactus.html*](https://sabutterflies.org.au/papi/anactus.html) (2024).
74. Byun, B. K. A review of the genus Spilonota Stephens (Lepidoptera: Tortricidae) in North Korea. *Entomol. Res.* **41,** 242–246 (2011).
75. Byun, B. K. & Yan, S. Check list of the tribe Tortricini (Lepidoptera: Tortricidae) in Northeast China, with two newly recorded species from China. *Korean Journal of Applied Entomology* **43,** 91–101 (2004).
76. Byun, B. K., Yan, S., Lee, B.-W. & Li, C.. Four newly recorded species of the genus Epinotia Hübner (Lepidoptera: Tortricidae) from Northeast China. *J. Asia-Pac. Entomol.* **11,** 181–184 (2008).
77. Byun, B. K., Yan, S. & Li, C. Revision of tribe Archipini (Tortricidae: Tortricinae) in Northeast China. *J. For. Res.* **14,** 93–102 (2003).
78. CABI International. CABI Compendium Datasheets <https://www.cabidigitallibrary.org/> (2024).
79. Cajiao, C., Rodríguez, O. & Pulido, J. I. Ciclo de vida y hábitos de Pococera atramentalis lederer (Pyralídae) plaga de la panoja del sorgo. *Acta Agronómica* **34**, 53–58 (1984).
80. Calvache, G., Franco, B. & Pedro, N. Manual de plagas de la palma de aceite en Colombia (No. L-0761). Centro de Investigación en Palma de Aceite, Cenipalma [Colombia] https://www.cabi.org/wp-content/uploads/Aldana-2010-Oil-palm-pest-manual.pdf (2010).
81. Campbell, R. W., & Youngs, L. C. (1978). *Douglas-fir Tussock Moth: An Annotated Bibliography*. vol. 68. Department of Agriculture, Forest Service, Pacific Northwest Forest and Canadian Forest Service collections [*https://apps-scf-cfs.rncan.gc.ca/collections/en*](https://apps-scf-cfs.rncan.gc.ca/collections/en) (2015).
82. Capinera, J. L. *Encyclopedia of Entomology* (Springer Science & Business Media, 2008).
83. Cappuccino, N. & Price, P. W. *Population dynamics: new approaches and synthesis* (Elsevier, 1995).
84. Castro Bobadilla, G., González, J., Hernández-Baz, F., Núñez Camargo, M. C. & Núñez Sánchez, Á. E. Macadamia integrifolia Maiden and Betche (Proteaceae), a New Host Plant Record for Automeris zozine (Druce 1886) from a Cloud Forest at Veracruz State, Mexico. *Southwest. Entomol.* **43,** 535–538 (2018).
85. Castroviejo, S. & Ibáñez, A. *Estudios sobre la biodiversidad de la región de Bahía Honda (Veraguas, Panamá)*. vol. 20. (Editorial CSIC-CSIC Press, 2005).
86. CBD-CHM KOREA. An integrated database on Korea’s biological resources [*https://www.cbd-chm.go.kr*](https://www.cbd-chm.go.kr) (2024).
87. Cech, R. & Tudor, G. *Butterflies of the East Coast: an observer’s guide* (Princeton University Press, 2005).
88. Chen, T., Dai, X. & Eiseman, C. A checklist of gymnosperm-feeding leafminers (Arthopoda, Insecta) in North America and Europe. *Biodivers. Data J.* **10**, (2022).
89. Cheng-Jin, Q., De-Wei, L. & Bing-Xin, Z. Bionomics of the Manchurian catalpa shoot borer, Sinomphisa plagialis (Lepidoptera: Pyralidae) in Shandong, China. *Bull. Entomol. Res.* **84**, 533–540 (1994).
90. Choi, S. Taxonomic note on Lemyra inaequalis (Butler)(Lepidoptera, Arctiidae) in Korea. *Entomol. Res.* **34**, 169–172 (2004).
91. Choi, S. Lemyra flammeola (Moore)(Lepidoptera, Arctiidae), new to Korea. *Entomol. Res.* **40**, 277–279 (2010).
92. Cibrián, T. D., Ebel, B. H. & Méndez, M. J. *Insectos de conos y semillas de las coníferas de México* (Secretaría de Agricultura y Recursos Hidráulicos/US Departament of Agriculture, Forest Service. South Eastern Forest Experiment Station, 1986).
93. Cibrián-Tovar, D. *Cone and seed insects of the Mexican conifers*. vol. 40. XF2006269758 (outheastern Forest Experiment Station, 1986).
94. Ciesla, W. *Forest entomology: a global perspective* (John Wiley & Sons, 2011).
95. Claflin, S. H. & Allen, D. C. Biology of Ancylis discigerana (Lepidoptera: Tortricidae). *Can. Entomol.* **113,** 265–270 (1981).
96. Clare, G. K. & Singh, P. Laboratory rearing of the raspberry bud moth, Heterocrossa rubophaga (Lepidoptera: Carposinidae) on blackberry. *N. Z. J. Zool.* **18**, 89–91 (1991).
97. Clarke, J. F. G. New North American species and new assignments in the genus Chionodes (Lepidoptera: Gelechiidae). *Journal of the Washington Academy of Sciences* **37**, 243–254 (1947).
98. Collins, C. W. *The oriental moth (Cnidocampa flavescens walk) and its control* (US Department of Agriculture, 1933).
99. Common, B. & Slater, E. *Moths of Australia* (Brill, 2023). <https://doi.org/https://doi.org/10.1163/9789004630468>
100. Commonwealth of Massachusetts. MassWildlife’s Natural Heritage & Endangered Species Program [www.mass.gov/nhesp](http://www.mass.gov/nhesp) (2024).
101. Comoglio, L. & Racheli, L. Biology of Automeris jucunda (Lepidoptera: Saturniidae, Hemileucinae) with taxonomic notes on A. hamata subgroup. *J. Insect Biodivers.* .vol. 4. <http://www.insectbiodiversity.org> (2016).
102. Comstock, J. A. Miscellaneous notes on western Lepidoptera. *Southern California Academy of Sciences* **36**, 111–124 (1937).
103. Comstock, J. H. & Comstock, A. B. *A Manual for the Study of Insects* (Comstock Publishing Company, 1901).
104. Cordell, C. E. *et al.* *Forest Nursery Pests* (USDA Forest Service, Agricultural Handbook No. 680, 1989).
105. Costa Lima, A. Insetos do Brasil. 8 Tomo. Coleópteros. 2a parte*. Escola Nacional de Agronomia, Série Didática* **10**, 1–323 (1953).
106. Coulson, R. N. & Witter, J. A. *Forest entomology: ecology and management* (John Wiley & Sons, 1984).
107. Covell Jr, C. v. Moths of Eastern North America. *Virginia Museum of Natural History, Martinsville* (2005).
108. Crabo, L. G. & Schmidt, B. C. A revision of Admetovis Grote, with the description of a new species from western North America (Noctuidae, Noctuinae, Hadenini). *ZooKeys* **788,** 167–181 (2018).
109. Crabo, L. & Hammond, P. C. A revision of Mesogona Boisduval (Lepidoptera: Noctuidae) for North America with descriptions of two new species. *Journal of Research on the Lepidoptera* **34**, 83–98 (1997).
110. Craighead, F. C. *Insect enemies of eastern forests* (US Government Printing Office, 1950).
111. Cranshaw, W. *Garden insects of North America: the ultimate guide to backyard bugs* (Princeton University Press, 2004).
112. Cullen, J., Julien, M. & McFadyen, R. *Biological control of weeds in Australia* (Csiro Publishing, 2012).
113. Damman, H. Oviposition behaviour and clutch size in a group-feeding pyralid moth, Omphalocera munroei. *The Journal of Animal Ecology* 193–204 (1991).
114. Danilevskiĭ, A. S. *Lepidopterous fauna of the USSR and adjacent countries: a collection of papers dedicated to professor Alexsandr Sergeevich Danilevskii* (Brill Archive, 1989).
115. Dantchenko, A., Sourakov, A. & Emmel, T. C. Egg Structure and Notes on Biology of Theclinae from Primorye, Russian Far East (Lepidoptera: Lycaenidae). *Holarct. Lepid.* **2,** 27–38 (1995).
116. Danthanarayana, W. & Kathiravetpillai, A. Studies on the Ecology and Causes of Outbreaks of Ectropis bhurmitra Wkr.(Geometridae), the Twig Catepillar of Tea in Ceylon. *J. Appl. Ecol.* **41,** 311–322 (1969).
117. Davis, D. R. A revision of the North American moths of the superfamily Eriocranioidea with the proposal of a new family, Acanthopteroctetidae (Lepidoptera) (1978) <https://doi.org/10.5479/si.00810282.251>.
118. Davis, D. R. & Stonis, J. R. *A revision of the New World plant-mining moths of the family Opostegidae (Lepidoptera: Nepticuloidea).* Smithsonian Institution Scholarly Press (2007) <https://doi.org/10.5479/si.00810282.625>
119. Davis, D. R. & Wagner, D. L. Biology and systematics of the Neotropical leafminer genus Eucosmophora (Lepidoptera: Gracillariidae). *Tropical Lepidoptera Research* 1–40 (2002).
120. Davis, J. J. The Citrus leaf roller Psorosticha zizyphi (Stnt.) in Queensland (Lepidoptera: Oecophoridae) (1965).
121. de Prins, J., & de Prins, W. Afromoths, online database of Afrotropical moth species (Lepidoptera). World Wide Web electronic publication <http://www.afromoths.net> (2021).
122. de Prins, J., & de Prins, W. Global Taxonomic Database of Gracillariidae (Lepidoptera). World Wide Web electronic publication <http://www.gracillariidae.net> (2024).
123. de Prins, W., & Steeman, C. Catalogue of the Lepidoptera of Belgium [*https://projects.biodiversity.be/lepidoptera*](https://projects.biodiversity.be/lepidoptera) (2024)
124. De Vlinderstichting <https://www.vlinderstichting.nl/> (2024).
125. De-Gregorio, J. J. P., Fernández, D. & Requena, E. Las especies catalanas del género Phycita Curtis, 1828 (Lepidoptera: Pyralidae: Phycitinae). *Heteropterus Revista de Entomología* **12,** 115-121 (2012).
126. Dekle, G. W. & Fasulo, T. R. “Azalea Caterpillar, Datana major Grote & Robinson (Insecta: Lepidoptera: Notodontidae): EENY137 IN294, 6 2000”. *EDIS* 2003 (14). Gainesville, FL.” <https://doi.org/10.32473/edis-in294-2000> (2003).
127. Delgado, C. & Couturier, G. Atteva punctella Cramer (Lepidoptera: Yponomeutidae), plaga de Simarouba amara Aubl.(Simaroubaceae), en Perú. *Boletin de la Sociedad Entomologica Aragonesa* **43**, 519–521 (2008).
128. Diakonoff, A. On some Indian Tortricidae (Lepidoptera). *Records of the Zoological Survey of India* **41**, 231–233 (1939).
129. Diakonoff, A. The south Asiatic Olethreutini (Lepidoptera, Tortricidae). Zoölogische Monographieën van het Rijksmuseum van Natuurlijke Historie 1:[i–xxii] 1–700. (1973).
130. Diakonoff, A. On a collection of some families of micro-Lepidoptera from Sri Lanka (Ceylon). *Zoologische Verhandelingen* **193**, 1–124 (1982).
131. Diakonoff, A. & Arita, Y. The early stages of Thaumatographa eremnotorna Diakonoff & Arita with remarks on the status of the Hilarographini (Lepidoptera Tortricoidea). *Entomologische Berichten* **41**, 56–60 (1981).
132. Dockter, D. E. Developmental changes and wear of larval mandibles in Heterocampa guttivitta and H. subrotata (Notodontidae). *J Lepid. Soc.* **47**, 32–48 (1993).
133. Duckworth, W. D. Neotropical Microlepidoptera XX: Revision of the genus Setiostoma (Lepidoptera: Stenomidae*).* In *Smithsonian Contributions to Zoology*, 106 (U.S. Government Printing Office, 1971).
134. Dueñas-López, M. A. *Lymantria xylina (casuarina tussock moth)*. CABI Compendium (2022) <https://doi.org/10.1079/cabicompendium.31815>.
135. Dugdale, J. S., Emmerson, A. W. & Hoare, R. J. B. Declana and Ipana (Insecta: Lepidoptera: Geometridae: Ennominae). *Fauna of New Zealand* **82**, 1–120 (2023).
136. Duncan, R. W. Common pitch moths of pine in British Columbia <https://publications.gc.ca/collections/collection_2012/rncan-nrcan/Fo29-6-69-1996-eng.pdf> (1996).
137. Duncan, R. W. (2006). *Conifer defoliators of British Columbia* (CFS, Pacific Forestry Centre, Victoria, B.C., 2006).
138. Dyar, H. G. Notes on drepanid larvae. *Journal of the New York Entomological Society* **3**, 66–69 (1895).
139. Eastman, J. A. *The book of forest and thicket: trees, shrubs, and wildflowers of eastern North America* (Stackpole Books, 1992).
140. Ebel, B. H. *Insects affecting seed production of slash and longleaf pines: Their identification and biological annotation*. vol. 6. (Southeastern Forest Experiment Station, 1963).
141. Eiseman, C. Buffaloberry miners. *VES News* **87**, 9–10 (2015).
142. Eiseman, C., Charney, N. & Carlson, J. *Tracks & sign of insects & other invertebrates: a guide to north American species* (Stackpole Books, 2010).
143. Eiseman, C. S. Notes on the larval habits and parasitoids of Rhopobota dietziana (Kearfott, 1907)(Tortricidae: Olethreutinae). *J. Lepid. Soc.* **68**, 218–220 (2014).
144. Eiseman, C. S. *et al.* A new species of Marmara (Lepidoptera: Gracillariidae: Marmarinae), with an annotated list of known hostplants for the genus. *Zootaxa* **4337** (2017).
145. Ellis, W. N. Plant Parasites of Europe. Leafminers, galls and fungi [*https://bladmineerders.nl/*](https://bladmineerders.nl/) (2024).
146. Emmel, T. C., Minno, M. C. & Drummond, B. A. *Florissant butterflies: a guide to the fossil and present-day species of central Colorado* (Stanford University Press, 1992).
147. Engelhardt, G. P. *The North American clear-wing moths of the family Aegeriidae* (US Government Printing Office, 1946).
148. EPPO Global Database <https://gd.eppo.int/> (2024).
149. Epstein, M. E. Biology of Dalcerides ingenita (Lepidoptera: Dalceridae). *Tropical Lepidoptera Research* **8,** 49–59 (1997).
150. Espy, M. & Babbitt, B. *Final supplemental environmental impact statement on management of habitat for late-successional and old-growth forest related species within the range of the northern spotted owl*. vol. 1 (US Department of Agriculture, Forest Service, 1994).
151. Estay, S. A. *Forest pest and disease management in Latin America: Modern perspectives in natural forests and exotic plantations* (Springer, 2020).
152. Estévez, A. A. Biología y morfología externa de los estadios inmaduros de Spodoptera dolichos (Lepidoptera: Noctuidae). *Novitates Caribaea* **17,** 59–70 (2021).
153. Fairweather, M. lou. *Field guide to insects and diseases of Arizona and New Mexico forests* (USDA Forest Service, Southwestern Region, 2006).
154. Falck, P., Karsholt, O. & Slamka, F. New data on Pyraloidea from the Canary Islands, Spain 2 (Lepidoptera: Pyralidae, Crambidae. *SHIL. Rev. lepidopterol.* **50**, 469–488 (2022).
155. Fauske, G. M. Moths of North Dakota: an online identification guide <https://www.ndsu.edu/ndmoths/ndmoths/> (2009).
156. Fenemore, P. G. *Plant pests and their control* (Butterworth-Heinemann, 2013).
157. Ferguson, D. C. *The Moths of America North of Mexico. Noctuoidea, Lymantriidae (Fascicle 22.2)* (Wedge Entomological Research Foundation, 1978).
158. Ferguson, D. C. *The Moths of America North of Mexico. Fascicle 18.1. Geometroidea, Geometridae (Part), Geometrinae* (Wedge Entomological Research Foundation, 1985).
159. Ferguson, D. C. A revision of the species of Nematocampa (Geometridae: Ennominae) occurring in the United States and Canada. *J. Lepid. Soc.* **47,** 60–77 (1993).
160. Ferguson, D. C. *Geometroidea: Geometridae (part), Ennominae (part-Abraxini, Cassymini, Macariini)* (Wedge Entomological Research Foundation, 2008).
161. Ferris, C. D. & Kruse, J. J. A new species of Zeiraphera Treitschke (Tortricidae). *J. Lepid. Soc*. **62,** 31 (2008).
162. Finnish Biodiversity Information Facility (FinBiF) <https://laji.fi/en> (2024).
163. Firake, D. M., Behere, G. T., Firake, P. D., Thakur, N. S. A. & Dubal, Z. B. An outbreak of pine lappet moth, Kunugia latipennis, in mid-altitude hills of Meghalaya state, India. *Phytoparasitica* **40**, 231–234 (2012).
164. Fitzgerald, T. D. Coexistence of three species of bark-mining Marmara (Lepidoptera: Gracillariidae) on green ash and descriptions of new species. *Annals of the Entomological Society of America* **66**, 457–464 (1973).
165. Fitzgerald, T. D. & Simeone, J. B. Serpentine miner Marmara fraxinicola (Lepidoptera: Gracillariidae) in stems of white ash. *Ann. Entomol. Soc. Am.* **64**, 770–773 (1971).
166. Flint, M. L. *Integrated pest management for almonds*. vol. 3308 (University of California Agriculture and Natural Resources, 2002).
167. Forbes, W. T. M. *The Lepidoptera of New York and neighboring states*. vol. 68. (Cornell University, 1923).
168. Forest Genetics Council of British Columbia. *“Cone and Seed Insect Pest Leaflet No.13”* [*https://forestgeneticsbc.ca/*](https://forestgeneticsbc.ca/) (2020).
169. Forest pests on the territories of the former USSR <http://www.eppo.org/QUARANTINE/forestry_project/EPPOforestry_project.pdf> (EPPO, 2005).
170. Forest Rocky Mountain and Range Experiment Station. *Large scale spatial patterns of conifer diseases in the Bighorn Mountains, Wyoming* (US Department of Agriculture, 1993).
171. Freeman, T. N. New Canadian species of leaf-mining Lepidoptera on conifers. *Journal of Research on the Lepidoptera* **4**, 209–220 (1965).
172. Furniss, R. L. *Western forest insects* (Washington, Department of Agriculture, Forest Service, 1977).
173. Furtado, E. & Lemaire, C. The biology and immature stages of Automeris granulosa (Lepidoptera: Saturniidae: Hemileucinae). *Tropical Lepidoptera Research* **10,** 27–29 (1999).
174. Furtado, E. & Racheli, L. The preimaginal stages of Othorene hodeva (Druce, 1904). *Neue Entomologische Nachrichten* **41,** 173–176 (1998).
175. Gadad, H., Naqvi, A. H., Mittal, V., Singh, J. & Das, S. Biology and Damage Pattern of Hairy Caterpillar Selepa celtis Moore (Lepidoptera: Nolidae) on Terminelia arjuna. *Int. J. Curr. Microbiol. Appl. Sci* **10**, 25–31 (2021).
176. Gall, L. F. Underwing Moths – The Genus Catocala (Lepidoptera: Noctuidae). in *Encyclopedia of Entomology* (ed. Capinera, J. L.) 4024–4026 (Springer Netherlands, 2008). <https://doi.org/10.1007/978-1-4020-6359-6_2297>.
177. Gall, L. F., Peacock, J. W. & Slotten, J. R. Life history and immature Stages of Catocala atocala (Noctuidae). *J. Lepid. Soc.* **56,** 1–4 (2002).
178. García-García, L. D. *et al.* Automeris moloneyi Druce Larvae Feed on Oil Palm Leaflets. *Southwest. Entomol.* **47**, 633–642 (2022).
179. Garijo, C. & García, E. J. Phyllocnistis citrella (Stainton, 1856)(Insecta: Lepidoptera: Gracillariidae: Phyllocnistinae) en los cultivos de cítricos de Andalucía (Sur España): biología, ecología y control de la plaga. *Bol. San. Veg. Plagas* **20**, 8 (1994).
180. Garnas, J., Hurley, B., Slippers, B., Wingfield, M. J. & Roux, J. in *Insects and diseases of Mediterranean forests* (Springer, 2016).
181. Gates, M. W. *et al.* Survey of the parasitic Hymenoptera on leafminers in California *J. Hym. Res*. **11,** 213–279 (2002).
182. Gelechiid Recording Scheme <https://gelechiid.co.uk/> (2024).
183. Gilligan, T. M., Baixeras, J. & Brown, J. W. T@RTS: Online World Catalogue of the Tortricidae (Ver. 4.0) <http://www.tortricid.net/catalogue.asp> (2018).
184. Gilligan, T. M. & Epstein, M. E. TortAI Tortricids of Agricultura Importance <https://idtools.org/id/leps/tortai> (2014).
185. Gonçalves, G. A. S., Barbosa, F. S. & Paluch, M. Biology and External Morphology of the Immature Stages of Dirphia moderata Bouvier (Lepidoptera: Saturniidae: Hemileucinae) in Anacardium occidentale L. *Braz. J. Biol.* **80**, 147–157 (2019).
186. Gorbunov, O. G. New data on clearwing moths (Lepidoptera: Sesiidae) of Sakhalin island. *Far East. Entomol.* **449**, 21–28 (2022).
187. Greatorex-Davies, N. Butterflies and Moths of Bulgaria <https://www.bulgarialeps.com/> (2024).
188. Green, A. J. The Sawflies (Symphyta) of Britain and Ireland <https://www.sawflies.org.uk/> (2021).
189. Grehan, J. R., Sabourin, M. & Hanson, P. M. *Maple feeding Tortricidae of the northeastern United States: guide to identification of adults* (Vermont Agricultural Experiment Station, 1995).
190. Grisales Vásquez, N. Y., Orozco Orozco, L. F., Giraldo Sánchez, C. E. & Pulgarín Díaz, J. A. First record of Phidotricha erigens (Lepidoptera: Pyralidae) feeding on Furcraea spp. in Colombia. *Revista Ceres* **68**, (2021).
191. Grund, R. “South Australian butterflies. Data sheet. Papilio demoleus sthenelus” <https://sabutterflies.org.au/files/datasheets/papi/Papilio%20demoleus%20sthenelus%20W.S.%20Macleay%20%20(Chequered%20Swallowtail).pdf> (2002).
192. Guiping, Y. & Bangnian, G. Biological characteristics and control of Pammene ginkgoicola. *Kun Chong zhi shi= Kunchong Zhishi* **41**, 475–477 (2004).
193. Gupta, R. & Tara, J. S. Biological studies of Lymantria obfuscata Walker (Lepidoptera: Lymantridae) on apple plantations (Malus domestica Borkh.) in Jammu region of J & K, India. *Munis Entomology & Zoology* **8**, 749–755 (2013).
194. Haack, R. A. Observations on Two Stem-Boring Coleoptera of Prickly-Ash (Zanthoxylum americanum) in Michigan: Micracis suturalis (Curculionidae: Scolytinae) and Sternidius alpha (Cerambycidae). *The Great Lakes Entomologist* **56**, 11 (2023).
195. Hagle, S. K. *Field guide to diseases and insect pests of northern and central Rocky Mountain conifers* (US Department of Agriculture, Forest Service, 2003).
196. Hain, F. P. & Wallner, W. E. The life history, biology, and parasites of the pine candle moth, *Exoteleia nepheos* (Lepidoptera: Gelechiidae), on scotch pine in Michigan. *Can. Entomol.* **105,** 157–164 (1973).
197. Hall, S. P. *Assessment of risk to non-target macro-moths after BTK application to Asian Gypsy Moth in the Cape Fear region of North Carolina*. vol. 98. (USDA, Forest Service, FHTET, 1999).
198. Hall, S. P. *et al.* Moths of North Carolina. Raleigh (NC): North Carolina Biodiversity Project and North Carolina State Parks. <https://auth1.dpr.ncparks.gov/moths/index.php>.
199. Hallman, G. J. & Knight Jr, R. J. Hypocala andremona (Lepidoptera: Noctuidae) development on eight species of Diospyros (Ebenaceae). *Fla. Entomol.* 461–465 (1993).
200. Han, H. L., LI, C. & Byun, B. New record of Trisuloides rotundipennis Sugi (Lepidoptera: Noctuidae: Pantheinae) from Korea. *Entomological Research* **37,** 36–38 (2007).
201. Handfield, L. & Handfield, D. A revision of the Canadian species of the Genus Herpetogramma Lederer, 1863 (Lepidoptera: Crambidae: Spilomelinae: Herpetogrammatini), with descriptions of three new species. *Bonn Zool. Bull.* **70**, 173–199 (2021).
202. Hanson, H. S. Ecological notes on the Sirex wood wasps and their parasites. *Bull. Entomol. Res.* **30**, 27–65 (1939).
203. Harada, M., Ohshima, Y., Yoshida, Y. & Wang, M. Report of surveys on the early stages of butterflies in the Nanling area (4). *Lepidoptera Science*, **63,** 165–174 (2012).
204. Harinath, P., Prasanna Kumar, V. & Venkata Ramana, S. P. Eco-biology of the Common Pierrot Castalius rosimon (Fabricius)(Lepidoptera: Rhopalocera: Lycaenidae). *World Journal of Zoology* **7**, 216–220 (2012).
205. Harris, M. K., Rogers, C. E. & Shanks, C. *The entomology of indigenous and naturalized Systems in Agriculture* (CRC Press, 2019).
206. Harrison, T. L. & Berenbaum, M. R. Anacampsis rhoifructella (Clemens): clarification of its identity and larval biology, and differentiation from a similar species, Anacampsis consonella (Zeller), revised status (Lepidoptera: Gelechiidae). *Zootaxa* **3794**, 545–555 (2014).
207. Hausmann, A. & Viidalepp, J. Systematic checklist of the Geometridae of Europe and adjacent areas .vol. 3. in *Larentinae I* 688–695 (Brill, 2012a).
208. Hausmann, A. & Viidalepp, J. *The geometrid moths of Europe*. vol. 3 (Apollo Books, 2012b).
209. Hawks, D. C. Review of the Catocala delilah species complex (Lepidoptera, Erebidae). *ZooKeys* **39**, 13–35 (2010).
210. Heather, N. W. Life history and biology of the leaf bagworm, Hyalarcta huebneri (Westwood)(Lepidoptera: Psychidae). *Aust. J. Entomol.* **14**, 353–361 (1976).
211. Hedlin, A. F. *et al.* *Cone and seed insects of North American conifers* (Canadian Forestry Service, 1981).
212. Heinrich, C. *Revision of the North American Moths of the Subfamily Eucosminae of the Family Olethreutidae: By Carl Heinrich*. (US Government Printing Office, 1923).
213. Heitzman, R. L. *Descriptions of the mature larva and pupa of Hypomecis umbrosaria (Hübner)(Lepidoptera: Geometridae)* (Entomological Society of Washington, 1982).
214. Henderson, C. L. *Butterflies, moths, and other invertebrates of Costa Rica: A field guide*. (University of Texas Press, 2010).
215. Heppner, J. B. Neomachlotica, a new genus of Glyphipterigidae (Lepidoptera). *Proc. Entomol. Soc. Wash.* **83,** 479–488 (1981).
216. Herbison-Evans, D. & Crossley, S. Caterpillars of Butterflies and Moths in Australia <https://lepidoptera.butterflyhouse.com.aul> (2024).
217. Hernández, Á. & Falcó, J. V. Quantitative parameters and ecological implications of a specialized tritrophic interaction involving a seed-feeding tortricid, Pseudargyrotoza conwagana, a braconid parasitoid, Bracon otiosus, and the wild privet, Ligustrum vulgare. *J. Insect Sci.* **14**, 128 (2014).
218. Herrick, G. W. *Insect enemies of shade-trees* (Concept Publishing Company, 1935).
219. Hetrick, L. A. Nepytia semiclusaria (Wlk.) as a defoliator of pine (Lepidoptera: Geometridae). *The Florida Entomologist* **43**, 205–206 (1960).
220. Hill, D. S. *Agricultural insect pests of the tropics and their control*. (Cambridge University Press, 1987).
221. Hill, D. S. *Pests of crops in warmer climates and their control* (Springer Science & Business Media, 2008).
222. Hill, M. G. Analysis of the biological control of Mythimna separata (Lepidoptera: Noctuidae) by Apanteles ruficrus (Braconidae: Hymenoptera) in New Zealand. *The Journal of Applied Ecology* **25,** 197–208 (1988).
223. Hiratsuka, Y., Cerezke, H. F., Petty, J. & Still, G. N. *Forest insect and disease conditions in Alberta, Saskatchewan, Manitoba, and the Northwest Territories in 1980 and predictions for 1981* (Canadian Forest Service, 1981).
224. Hirowatari, T., Yagi, S., Ohshima, I., Huang, G.-H. & Wang, M. Review of the genus Vespina (Lepidoptera, Incurvariidae) with two new species from China and Japan. *Zootaxa* **4927**, 209–233 (2021).
225. Hoare, R. J. B. Hierodoris (Insecta: Lepidoptera: Gelechioidea: Oecophoridae). In: *Fauna of New Zealand* (Manaaki Whenua Press, 2005).
226. Hoare, R. J. B. Noctuinae (Insecta: Lepidoptera: Noctuidae). Part 1, Austramathes, Cosmodes, Proteuxoa, Physetica. *Fauna of New Zealand* **73** (2017).  <https://doi.org/10.7931/J2/FNZ.73>.
227. Hodges, R. W. *The Moths of America North of Mexico: Fascicle 6.1-Gelechioidea, Cosmopterigidae* (Wedge Entomological Research Foundation, 1978).
228. Hodges, R. W. *The Moths of America North of Mexico: Fascicle 7.1-Gelechioidea, Gelechiidae* (Wedge Entomological Research Foundation, 1986).
229. Hodges, R. W. & Stevens, R. E. Two new pine-feeding species of Coleotechnites (Gelechiidae). *J. Lepid. Soc.* **32,** 118–122(1978).
230. Hoffmann, F. Beiträge zur Naturgeschichte brasilianischer Schmetterlinge. *Berliner entomologische Zeitschrift* **1932**, 97–148 (1932).
231. Hokkaido Pest Control Center <http://www.agri.hro.or.jp/> (2024)
232. Hope, J. H. & Pless, C. D. Biology of Euzophera ostricolorella on Yellow-Poplar in Tennessee. *Ann. Entomol. Soc. Am.* **72**, 1–4 (1979).
233. Hopkins, A. D. *et al.* *Catalogue of exhibits of insect enemies of forests and forest products at the Louisiana Purchase Exposition, St. Louis, Mo., 1904*. vol. 42 (US Department of Agriculture, Division of Entomology, 1903).
234. Howard, F. W., Giblin-Davis, R., Moore, D. & Abad, R. *Insects on palms* (Cabi, 2001).
235. Hoyle, P. C. Pococera atramentalis Led.(Perforador del ápice de la bellota del algodonero) y la reglamentación del cultivo de maíz. *Revista Peruana de Entolomogía* **4**, 78–81 (1961).
236. Hrudová, E. Nontarget. Lepidoptera species found in the pheromone traps for selected tortricid species in 2002 and 2003 years. *Acta Universitatis Agriculturae et Silviculturae Mendelianae Brunensis* **53**, 35–44 (2005).
237. Hsu, Y.-F. & Lin, M.-Y. Systematic position of Sibataniozephyrus and description of a new species from Taiwan (Lycaenidae: Theclinae). *J. Lepid. Soc.* **48**, 128–147 (1994).
238. Huemer, P. Der Teleiodes vulgella-Artenkomplex (Lepidoptera: Gelechiidae). *Zeitschrift der Arbeitsgemeinschaft Österreichischer Entomologen* **44**, 1–14 (1992).
239. Huemer, P. & Karsholt, O. *Gelechiidae II:(Gelechiinae: Gnorimoschemini)*. vol. 6. (Brill, 2013).
240. Huemer, P. & Sattler, K. A taxonomic revision of palaearctic Chionodes (Lepidoptera: Gelechiidae). *Beitr. Ent.* **45,** 3–108 (1995).
241. Illustrated guide of butterfly moths produced in Taiwan. WWW publications <http://dearlep.tw> (2024).
242. Indo-China Butterfly check list. Chiefly from Thailand, Laos & Vietnam <https://yutaka.it-n.jp/> (2024).
243. Indian Biodiversity Portal. Species data <https://indiabiodiversity.org/> (2022).
244. Inoue, H. A revision of the Japanese Lymantriidae (II). *Jpn. J. Med. Sci. Biol.* **10**, 187–219 (1957).
245. Insects of Tokyo: Lepidoptera <http://tokyoinsects.web.fc2.com/tokyo_lepidoptera.html> (2006).
246. Institute of Food and Agricultural Sciences - University of Florida (UF/IFAS). Featured Creatures. Entomology & Nematology <https://entnemdept.ufl.edu/creatures/> (2024)
247. Jakšić, P. New contributions to the knowledge of Lepidortera fauna of Kosovo and Metohia (Republic of Serbia). *Bull. Nat. Sci. Res.* **6**, 1–4(2016).
248. James, A. Biology of Polygonia progne nigrozephyrus and related taxa (Nymphalidae). *J. Lepid. Soc.* **42**, 46–56 (1988).
249. James, D. G. *The book of caterpillars: a life-size guide to six hundred species from around the world* (University of Chicago Press, 2017).
250. Janzen, D. H. Two ways to be a tropical big moth: Santa Rosa saturniids and sphingids. *Oxford Surveys in Evolutionary Biology* **1,** 85–140 (1984).
251. Jessen, E. Life History of the Monterey Pine Needle Miner, Argyresthia pilatella (Lepidoptera: Yponomeutidae). *Ann. Entomol. Soc. Am.* **57**, 332–341 (1964).
252. Jiazhi, L., Guocai, Z. & Hongzhi, S. Chemical control of Zethenia rufescentaria Motsch. *J. Northeast For. Univ.* **7**, 28–31 (1996
253. Jinbo, U. A systematic study of the pulchra species group of Archips occurring in Japan (Lepidoptera, Tortricidae). *Entomol. Sci.* **9**, 327–340 (2006).
254. Johns, C. A., Tangalin, N., Bustamente, K. & Kawahara, A. Y. Evidence of an undescribed, extinct Philodoria species (Lepidoptera: Gracillariidae) from Hawaiian Hesperomannia herbarium specimens. *Proceedings of the Hawaiian Entomological Society* (2014).
255. Jones, J. R., DeByle, N. v. & Bowers, D. M. *Insects and other invertebrates in Aspen: Ecology and Management in the Western United States*. General Technical Report RM-119 (USDA Forest Service, 1985).
256. Journal of agricultural research. vol*.*27*.* no.1-13 <https://www.biodiversitylibrary.org/item/280953> (U.S. Govermnet Printing Office, 1924).
257. Journal of agricultural research. *Vol.30. no.1-12 (1925)*. <https://www.biodiversitylibrary.org/item/280991> (U.S. Govermnet Printing Office, 1925).
258. Karowe, D. N. Diapause dynamics and host plant utilization of Colias philodice, Colias interior and their hybrids (Lepidoptera: Pieridae). *The Great Lakes Entomologist* **27**, 3 (1994).
259. Kasy, F. Schmetterlingsfauna des Naturschutzgebietes Hackelsberg, Nordburgenland*. Z. ArbGem. ost. Ent.* **30,** 1–44 (1978).
260. Keen, F. P. *Insect enemies of western forests*. (US Government Printing Office, 1952).
261. Keen, F. P. *Cone and seed insects of western forest trees*. (US Department of Agriculture, 1958).
262. Keifer, H. H. California microlepidoptera VIII. *Bulletin of the Southern California Academy of Sciences* **35**, 9–29 (1936).
263. Khan, S. A., Bhatia, S. & Tripathi, N. Entomological studies of chaetoprocta odata, an important pest on walnut trees (Juglans Regia L.) in Kashmir valley. *J. Acad. Ind. Res.* **2**, 378–381 (2013).
264. Khewa, S. & Mukhopadhyay, A. Biocontrol potential of a newly isolated bacterial agent against Arctornis submarginata (Walker)(Lepidoptera: Lymantriidae) occurring in Darjeeling Terai region. *J. Biopestic.* **3**, 114 (2010).
265. Kim, M. J., Kim, S.-S., Choi, S.-W. & Kim, I. Saturnia jonasii Butler, 1877 on Jejudo Island, a new saturnid moth of South Korea with DNA data and morphology (Lepidoptera: Saturniidae). *Zootaxa* **3946**, 374–386 (2015).
266. Kim, N.-H., Sohn, J.-C. & Choi, S.-W. Larvae and host plants of ten lepidopteran species from Mt. Jirisan (South Korea) with two species of Tortricidae new to Korea. *Japan Heterocerists’ Journal* **275**, 634–637 (2015).
267. Kimber, I. UKmoths. Online guide to the moths of Great Britain and Ireland <https://ukmoths.org.uk/> (2024)
268. Kimsey, L. S., Gilligan, T. M., Carpenter, J. M., Smith-Pardo, A. H. & Redford, A. J. Identification Technology Program (ITP). North American Hornet Screening Tool <https://idtools.org/hornet_screening/> 2021.
269. Kirk, K. *Conservation Assessment for Henry’s Elfin Butterfly (Callophrys henrici)* (USDA Forest Service, Eastern Region, 2003).
270. Kishida, Y. *Standard Illustrated Guide to Japanese Moths 2*. (Gakken Educational Publishing, 2007).
271. Kitching, R. L. *Biology of Australian butterflies*. vol. 6. (Csiro Publishing, 1999).
272. K.L., N., & Ochoa, I. *Insects and mites associated with Ontario forests: Classification, common names, main hosts and importance* <https://publications.gc.ca/collections/collection_2008/nrcan/Fo123-2-7-2006E.pdf> (CFS Canadian Forest Service, 2006).
273. Kliejunas, J. T. *Pest risk assessment of the importation into the United States of unprocessed Eucalyptus logs and chips from South America*. vol. 124. (US Department of Agriculture, Forest Service, Forest Products Laboratory, 2001).
274. Klinkenberg, Brian. (Ed. ). E-Fauna BC: Electronic Atlas of the Fauna of British Columbia Lab for Advanced Spatial Analysis, Department of Geography, University of British Columbia, Vancouver [*www.efauna.bc.ca*](http://www.efauna.bc.ca) *(2021).*
275. Konno, K. *et al.* GABA, β-alanine and glycine in the digestive juice of privet-specialist insects: convergent adaptive traits against plant iridoids. *J. Chem. Ecol.* **36**, 983–991 (2010).
276. Kononenko, V. S. & Behounek, G. A revision of the genus Lophomilia Warren, 1913 with description of four new species from East Asia (Lepidoptera: Noctuidae: Hypeninae). *Zootaxa* **1989**, 1–22 (2009).
277. Kooster, S. & Sinev, S. *Momphidae sl:(Momphidae, Batrachedridae, Stathmopodidae, Agonoxenidae, Cosmopterigidae, Chrysopeleiidae)*. vol. 5. (Brill, 2021).
278. Kopelke, J.-P. The host plants of Euura cinereae Kopelke, 1996 and E. auritae Kopelke, 2000 (Hymenoptera: Tenthredinidae). *Entomol. Fenn.* **13**, 134–138 (2002).
279. Korshunov, Y. P., & Gorbunov, P. Y. Butterflies of the Asian part of Russia <https://pisum.icgbio.ru/kosterin/korgor/index.htm> (1995)
280. Kudo, G. Variations in leaf traits and susceptibility to insect herbivory within a Salix miyabeana population under field conditions. *Plant Ecol.* **169**, 61–69 (2003).
281. Kumar, P. & Negi, P. S. Records of Lepidopteron Borers (Lepidoptera) on Stored Seeds of Indian Himalayan Conifers. *International Journal of Bioengineering and Life Sciences* **12**, 333–336 (2018).
282. Kumata, T. Taxomonic studies on the Lithocolleinae of Japan (Lepidoptera: Gracillariidae) Part 1. *Insecta matsumurana* **25**, 53–90 (1963a).
283. Kumata, T. Taxonomic studies on the Lithocolletinae of Japan (Lepidoptera: Gracillariidae) Part 2. *Insecta Matsumurana*, **26,** 1–48 (1963b).
284. Kumata, T. Descriptions of twenty new species of the genus Caloptilia Hübner from Japan including the Ryukyu Islands (Lepidoptera: Gracillariidae). *Insecta Matsumurana* **29**, 1–21 (1966).
285. Kumata, T. On the Japanese species of the genera Macarostola, Aristaea and Systoloneura, with descriptions of three new species (Lepidoptera: Gracillariidae). *Insecta matsumurana* **9**, 1–51 (1977).
286. Kumata, T. A taxonomic revision of the Gracillaria group occurring in Japan (Lepidoptera: Incurvarioidea). *Insecta matsumurana* **26**, 1–186 (1982).
287. Kumata, T., Kuroko, H. & Ermolaev, V. P. Japanese species of the Acrocerocops-group (Lepidoptera: Gracillariidae). Part 1. *Insecta Matsumurana.* **38,** 1-111 (1988a).
288. Kumata, T., Kuroko, H. & Ermolaev, V. P. Japanese species of the acrocercops-group (Lepidoptera: Gracillariidae) part 2. *Insecta Matsumurana* **40,** 1–133 (1988b).
289. Kunte, K. *India, a Lifescape: butterflies of peninsular India* (Universities Press, 2000).
290. Kunte, K., Sondhi, S. & Roy, P. Butterflies of India, v. 4.16. Indian Foundation for Butterflies <https://www.ifoundbutterflies.org> (2014)
291. Kuroko, H. & Gaedike, R. Epermeniidae of Japan (Lepidoptera, Epermenioidea), with descriptions of six new species. *Lepidoptera Science* **57,** 49–69 (2006).
292. Kuroko, H. (1964). *Revisional studies on the family lyonetiidae of japan (Lepidoptera).* Kyushu University Institutional Repository <https://catalog.lib.kyushu-u.ac.jp/opac_download_md/2349/1.pdf> (1964).
293. Lafontaine, J. D. *The Moths of America North of Mexico, including Greenland. Fascicle 27.3. Noctuoidea: Noctuidae (Part): Noctuinae (Part--Noctuini)*. (Wedge Entomological Research Foundation, 1998).
294. Lafontaine, J. D. & Walsh, J. B. A revision of the genus Ufeus Grote with the description of a new species from Arizona (Lepidoptera, Noctuidae, Noctuinae, Xylenini, Ufeina). *ZooKeys* **193** (2013).
295. Lang, S. Y. Study on the tribe Chalingini Morishita, 1996 (Lepidoptera, Nymphalidae, Limenitinae). *Far East. Entomol.* **28,** 1–7 (2010).
296. Lawrence, P. O. The Jamaican’Orange Dog’, Papilio andraemon (Lepidoptera: Papilionidae). *Fla. Entomol.* **55,** 243–246 (1972).
297. Layberry, R. A., Hall, P. W. & Lafontaine, J. D. *The butterflies of Canada* (University of Toronto Press, 1998).
298. Lee, C.-M., Arita, Y. & Bae, Y.-S. A Newly Recorded Species of the Genus Milisipepsis (Lepidoptera, Sesiidae) from Korea. *Korean J. App. Entomol.* **50**, 363–365 (2011).
299. Lee, C.-M., Bae, Y.-S. & Arita, Y. Morphological description of Synanthedon bicingulata (Staudinger, 1887) in life stages (Lepidoptera, Sesiidae). *J. Asia-Pac. Entomol.* **7**, 177–185 (2004).
300. Lee, S. Three new species of the genus Pseudotelphusa Janse, 1958 (Lepidoptera: Gelechiidae) from North America. ). *J. Asia-Pac. Entomol.* **14**, 299–303 (2011).
301. Lee, S. & Brown, R. L. Review of the genus Sinoe (Lepidoptera: Gelechiidae) with descriptions of two new species. *Zootaxa* **3332**, 49–59 (2012).
302. Lee, Y. J. Apaturinae (Lepidoptera: Nymphalidae) from the Korean Peninsula: synonymic lists and keys to tribes, genera and species. *Zootaxa* **2169**, 1–20 (2009).
303. Leen, R. Biology of Uresiphita reversalis(Guenee) and comparison with U. polygonalis maorialis(Felder)(Crambidae). *J. Lepid. Soc.* **49**, 163–170 (1995).
304. Leininger, T. D. *A guide to major insects, diseases, air pollution injury, and chemical injury of sycamore*. vol. 28. (Southern Research Station, 1999).
305. Lelej, A. *Annotated catalogue of the Insects of Russian Far East. Volume II. Lepidoptera.* (Vladivostok: Dalnauka, 2016).
306. Lemaire, C. Description d’u genre nouveau de République Dominicaine (Lepidoptera, Saturnüdae, Hemileucinae). *Atalanta* **30,** 177–178 (1999).
307. Leong, T. M. Last instar caterpillar and metamorphosis of Neostauropus alternus (Walker)(Lepidoptera: Notodontidae). *Nature in Singapore* **1**, 159–164 (2008).
308. Leraut, P. Contribution à l’étude des Archiearis Hübner et genres apparentés (Lepidoptera, Geometridae). *Bulletin de la Société entomologique de France* **107**, 349–358 (2002).
309. Li Junyan, W. X. *Taiwan Butterfly Illustrated Book* (2021).
310. Li, Y., Oguchi, S. & Goto, M. Physiology of diapause and cold hardiness in overwintering pupae of the apple leaf miner Phyllonorycter ringoniella in Japan. *Physiol. Entomol.* **27**, 92–96 (2002).
311. Liang, J. J. & Lee, M.-J. Status of forest trees infested with Endoclita sinensis (Lepidoptera: Hepialidae). *Taiwan J. For. Sci.* **26**, 203–210 (2011).
312. Liston, A. D., Savina, H., Nagy, Z. T., Sonet, G. & Boeve, J.-L. Taxonomy, phylogeny and host plants of some Abia sawflies (Hymenoptera, Cimbicidae). *Zootaxa* **3821**, 125–132 (2014).
313. Lizhong, W., Yuguang, H., Weimin, Z., Junli, J. & Ruili, G. Study on the bionomics of Epicopeia mencia and its control. *Forest Pest and Disease* **2**, 38–39 (1998).
314. Lotts, K. & Naberhaus, T. Butterflies and Moths of North America (BAMONA). Data set <http://www.butterfliesandmoths.org/> (2024).
315. Lv, D. *et al.* Larval midgut protease activity of Illiberis pruni (Lepidoptera: Zygaenidae) feeding on multiple characteristic hosts. *Phytoparasitica* **50**, 1033–1042 (2022).
316. Ma, J. S., Huang, Y. P., Pu, Y. H. & Fan, S. H. Today’s hometown of Metasequoia—Lichuan, Hubei, China. *Thaiszia* **14**, 23–36 (2004).
317. Maier, C. T. Life cycle of Coptodisca negligens (Lepidoptera: Heliozelidae) on cranberry. *J. Econ. Entomol.* **81**, 497–500 (1988).
318. Maier, C. T., Lemmon, C. R., Fengler, J. M., Schweitzer, D. F. & Reardon, R. C. *Caterpillars on the foliage of conifers in the Northeastern United States (Revised)* (Forest Health Technology Enterprise Team, 2011).
319. Malabika Kakati, M. K., Jatin Kalita, J. K. & Saikia, P. K. Biology of common leopard butterfly Phalanta phalanta (Lepidoptera) in Assam*. Journal of Ecobiology* **17,** 383–392(2005).
320. Mamay, M. *et al.* Infestation rate and cardinal directional preference of pistachio twig borer [Kermania pistaciella Amsel.(Lepidoptera: Tineidae)]. *Journal of King Saud University-Science* **34**, 102025 (2022).
321. Mani, M., Krishnamoorthy, A. & Ramanujam, B. Trends in the Biological Control of Horticultural Crop Pests in India. *Trends in Horticultural Entomology* 243–281 (2022).
322. Marini, M. & Russo, I. Interessanti reperti di Lepidotteri in Calabria. *Bollettino dell’Istituto di Entomologia dell’Università di Bologna* **35**, 249–265 (1980).
323. Markin, G. P. Notes on the biology and release of Caloptilia sp. nr. schinella (Walsingham)(Lepidoptera: Gracilariidae), a biological control moth for the control of the weed firetree (Myrica faya Aiton) in Hawaii*. Proc. Hawaii. Entomol. Soc*. **35,** 67–76 (2001).
324. Marquis, R. J. *et al.* *Illustrated guide to the immature Lepidoptera on oaks in Missouri* (United States Department of Agriculture, 2019).
325. Martín, L. O. A. *Las mariposas diurnas de Castilla y León:(Lepidópteros Ropalóceros): especies, biología, distribución y conservación*. (Junta de Castilla y León, Consejería de Medio Ambiente, 2007).
326. Mazzuferi, V. Plagas en Prosopis. *Multequina* **9**, 107–117 (2000).
327. Mbata, K. J. & de Prins, J. Annotated checklist of moths of Zambia (Insecta: Lepidoptera). *Zootaxa* **5354**, 1–503 (2023).
328. McCabe, T. L. The larva of Acronicta spinigera Guenee (Noctuidae). *J. Res. Lepid.* **17**, 173–179 (1978).
329. McCabe, T. L. Hosts, biology, and distribution of Zale phaeocapna(Noctuidae). *J. Lepid. Soci.* **41**, 195–198 (1987).
330. McCormac, J. & Gottfried, C. *Gardening for Moths: A Regional Guide* (Ohio University Press, 2023).
331. McDunnough, J. H. *Revision of the North American species of the genus Eupithecia (Lepidoptera, Geometridae).* Bulletin of the AMNH. v. 93, article 8 (1949).
332. McGuffin, W. C. Larvae of the Nearctic Larentiinae (Lepidoptera: Geometridae). *The Memoirs of the Entomological Society of Canada* **90**, 5–104 (1958).
333. McPartland, J. M., Clarke, R. C. & Watson, D. P. *Hemp diseases and pests: management and biological control: an advanced treatise* (CABI, 2000).
334. Medvedev, G. S. *Keys to the Insects of the European Part of the USSR.: Lepidoptera. IV, 1*. vol. 117 (Brill Archive, 1989).
335. Mehrnejad, M. R. Arthropod pests of pistachios, their natural enemies and management. *Plant Prot. Sci.* **56**, 231–260 (2020).
336. Mehrnejad, M. R. & Mirzaei, R. Life cycle, damage and control of the pistachio bud-borer moth Telphusa pistaciae (Lepidoptera: Gelechiidae). *Appl. Entomol. Phytopathol.* **83**, 201–208 (2016).
337. Meierotto, S. *et al.* A revolutionary protocol to describe understudied hyperdiverse taxa and overcome the taxonomic impediment. *Deutsche Entomologische Zeitschrift* **66**, 119–145 (2019).
338. Meijerman, L., & Ulenberg, S. A. Arthropods of Economic Importance: Eurasian Tortricidae <https://eurasian-tortricidae.linnaeus.naturalis.nl> (2000).
339. *Memoirs of the National Academy of Sciences. vol.7* Government Printing Office. <https://www.biodiversitylibrary.org/page/6945597> (1985(.
340. Merrill, A. L. *Agriculture Handbook*. vol. 1. (US Department of Agriculture, 1949).
341. Michigan Natural Features Inventory. MSU Extension. Rare Species Explorer <https://mnfi.anr.msu.edu/species/explorer> (2024).
342. Miller, J. C. *Caterpillars of Pacific Northwest forests and woodlands*. vol. 6 (National Center of Forest Health Management, US Department of Agriculture, 1996).
343. Miller, J. C. *Macromoths of northwest forests and woodlands*. vol. 98. (Forest Health Technology Enterprise Team, USDA Forest Service, 2000).
344. Miller, J. C., & Cronhardt, J. E. Life history and seasonal development of the western winter moth, operophtera occidentals (Lepidoptera: geometridae), in Western Oregon. *Can. Entomol.* **114,** 629–636 (1982).
345. Miller, J. C. & Hammond, P. C. *Lepidoptera of the Pacific Northwest: caterpillars and adults*. (Forest Health Technology Enterprise Team, US Department of Agriculture …, 2003).
346. Miller, J. C., Janzen, D. H. & Hallwachs, W. *100 butterflies and moths: Portraits from the tropical forests of Costa Rica* (Harvard University Press, 2007).
347. Miller, W. E. Biology of Anacampsis innocuella (Zeller), a Leafroller on Aspen1. *J. Econ. Entomol.* **48**, 622–623 (1955).
348. Miller, W. E. Petrova houseri, a new pitch-nodule moth from eastern North America. *The Ohio J. Sci.* **59,** 230 (1959).
349. Miller, W. E. The species of Pseudexentera (Tortricidae). *J. Lepid. Soc.* **40,** *218*–*237* (1986).
350. Miller, W. E. *Guide to the Olethreutine moths of midland North America (Tortricidae)* (US Department of Agriculture, Forest Service, 1987).
351. Miller, W. E. Body size and diet quality in the genus Cydia (Tortricidae). *J. Lepid. Soc.* **44,** 113–142 (1990).
352. Miller, W. E. & Altmann, S. A. Ecological observations on the Virginia pitchnodule moth, Petrova wenzeli (Kearfott), including a note on its nomenclature (Lepidoptera, Olethreutidae). *Ohio J. Sci.* **58,** 273–281 (1958).
353. Miranda, A. v *et al.* The Saturniidae (Lepidoptera) deposited in the entomological collection of the Oswaldo Cruz Institute, Oswaldo Cruz Foundation, Rio de Janeiro, Brazil, with geographic and taxonomic notes. *Nachrichten des Entomologischen Vereins Apollo, NF* **36**, 136–147 (2015).
354. Mitra, S. *Guava: botany, production and uses* (CABI, 2021).
355. Mizell, R. F. The Pecan Bud Moth: Pest of Nursery Stock and Transplanted Pecans. *EDIS* **2004**, (2004).
356. Mizell, R. F. The Persimmon Borer Sannina uroceriformis Walker, Pest of Persimmon: ENY-835/IN669, 7/2006. *EDIS* **2006**, (2006).
357. Molina, J. M. Palatabilidad de las hojas del arándano americano (Vaccinium spp.) para Spodoptera littoralis (Boisduval, 1833)(Lepidoptera: Noctuidae). *Bol. San. Veg. Plagas* **26**, 89–98 (2000).
358. Mollaei, M., Izadi, H., Šimek, P. & Koštál, V. Overwintering biology and limits of cold tolerance in larvae of pistachio twig borer, Kermania pistaciella. *Bull. Entomol. Res.* **106**, 538–545 (2016).
359. Mooney, K. A. The life history of Dasypyga alternosquamella Ragonot (Pyralidae) feeding on the Southwestern dwarf mistletoe (Arceuthobium vaginatum) in Colorado. *J. Lepid. Soc.* **55**, 140–143 (2001).
360. Morris, O. N. Insect disease survey in British Columbia 1964-1969 (Canadian Forest Service (CFS) Publications, 1970).
361. Moulds, M., Tuttle, J. & Lane, D. *Hawkmoths of Australia: identification, biology and distribution*. vol. 13. (Csiro Publishing, 2020).
362. Muniappan, R. *Arthropod pests of horticultural crops in tropical Asia* (CABI, 2012).
363. Munyaneza, J. E. *Plan de contingencia de Xanthomonas citri pv. aurantifolii (Schaad et al.) Constantin et al. y Xanthomonas citri pv. citri (Hasse) Constantin et al. causantes de Citrus Canker (cancrosis de los cítricos).* Programa nacional para la aplicación de la normativa fitosanitaria (Ministerio de Agricultural, Pesca y Alimentación, 2020)
364. Catalan Butterfly Monitoring Scheme. Museu de Ciències Naturals de Granollers - Departament de Territori i Sostenibilitat de la Generalitat de Catalunya <https://mcng.cat/lepidopters-recerca/projectes-lepidopters/cbms-catalan-butterfly-monitoring-scheme> (2024).
365. Myburgh, A. C. & Basson, S. G. Tortnx capensana (Wlk.) and Epichorista ionephela (Meyr.) as pests new to apples and pears (Lepidopt.: Tortricidae).  *Journal of the Entomological Society of South Africa* **24,** 348–349 (1961).
366. Nair, N. *et al.* Insect pest complex of Pigeon pea (Cajanus cajan) in agro ecosystem of Tripura, NE India. *J. Entomol. Zool. Stud.* **5**, 765–771 (2017).
367. Nakamura, M., Asanuma, M. & Hiura, T. Differential effects of host plant hybridization on herbivore community structure and grazing pressure on forest canopies. *Oikos* **119**, 1445–1452 (2010).
368. NARO. National Agriculture and Food Research Organization. Fact Sheet “Antivaleria viridimacula (Graeser) Order: Lepidoptera” <https://www.naro.go.jp/english/> (2024).
369. Nässig, W. A. Zur Präimaginalmorphologie von Saturnia (Rinaca) zuleika und S.(R.) thibeta sowie Anmerkungen zu den Salassinae (Lepidoptera: Saturniidae). *Nachrichten des Entomologischen Vereins Apollo NF* **15**, 409–438 (1994).
370. Nasu, Y. A New Species and a Newly Recorded Species of Epinotia HUEBNER (Lepidoptera: Tortricidae) Injurious to Spruce in Japan. *Appl. Entomol. Zool.* **26**, 343–346 (1991).
371. Naves, P., Nóbrega, F. & de Sousa, E. Annual flight activity of Dioryctria mendacella (Lepidoptera: Pyralidae) in southern Portugal. *Phytoparasitica* **51**, 41–48 (2023).
372. Neal Jr, J. W. Bionomics and instar determination of Synanthedon rhododendri (Lepidoptera: Sesiidae) on rhododendron. *Ann. Entomol. Soc. Am.* **77**, 552–560 (1984).
373. Negrón-Ortiz, V. & Gorchov, D. L. Effects of fire season and postfire herbivory on the cycad Zamia pumila (Zamiaceae) in slash pine savanna, Everglades National Park, Florida. *Int. J. Plant Sci.* **161**, 659–669 (2000).
374. Nelson, M. W. (2019). *“Phyllira Tiger Moth Apantesis phyllira”.* [*https://www.mass.gov/doc/phyllira-tiger-moth/download*](https://www.mass.gov/doc/phyllira-tiger-moth/download)*.*
375. Nemer, N., Bal, J., Bechara, E. & Frérot, B. Pheromone identification of the cedar shoot moth Dichelia cedricola Diakonoff (Lepidoptera: Tortricidae). in *Annales de la Société entomologique de France (NS)* vol. 50 367–371 (Taylor & Francis, 2014).
376. Neunzig, H. H. *Taxonomy of Acrobasis larvae and pupae in Eastern North America (Lepidoptera: Pyralidae)*. Technical Bulletin No. 1457. Agricultural Research Service. (United States Department of Agriculture in cooperation with North Carolina Agricultural Experiment Station, 1972).
377. Neunzig, H. H. *The Moths of America North of Mexico. Fascicle 15.2. Pyraloidea, Pyralidae (part), Phycitinae (part--Acrobasis and allies)* (Wedge Entomological Research, 1986).
378. Neunzig, H. H. Last stage larva and pupa of Glyptocera consobrinella (Zeller)(Pyralidae: Phycitinae). *J. Lepid. Soc.* **45,** 112-116 (1991).
379. Neunzig, H. H. *The moths of America North of Mexico, Fascicle 15.5: Pyraloidea, Pyralidae, Phycitinae (part)* (Wedge Entomological Research Foundation, 2003).
380. Ng, P. K. L., Corlett, R. & Tan, H. T. W. *Singapore biodiversity: an encyclopedia of the natural environment and sustainable development* (Editions Didier Millet, 2011).
381. Noreika, Rem., & Puplesis, R. *Salicaceae feeding Gracillariidae (Lepidoptera) of Central Asia*. Zoologijos katedra VPI (1992).
382. Nozawa, A. & Inari, N. Abundance of inflorescence tissue feeders on three willow species. *Entomol. Sci.* **8**, 9–15 (2005).
383. New Zealand Farm Forestry NZFM. Association Inc. Pests and diseases of forestry in New Zealand <https://www.nzffa.org.nz/farm-forestry-model/the-essentials/forest-health-pests-and-diseases/forestry-diseases/> (2024).
384. O'Brien, Mark F. *Caterpillars of Eastern Forests* (USDA Forest Service FHTET-96-34, 1997).
385. Oehlke, B. The World’s Largest Saturniidae Site <http://www.silkmoths.bizland.com/> (2024)
386. Oei-Dharma, H. P. *Use of pesticides and control of economic pests and diseases in Indonesia* (Brill Archive, 1969).
387. Ogard, P. H. *Butterflies of Alabama: glimpses into their lives* (University of Alabama Press, 2017).
388. Oku, T. Descriptions of nine new species of the genus Coleophora from Japan, with notes on other species (Lepidoptera : Coleophoridae). *Insecta matsumurana* **27**, 114–124.
389. Olson, C. E. *50 Common Insects of the Southwest* (Western National Parks Association, 2004).
390. Opler, P. A. Seven new lepidoterous leaf-miners associated with Quercus agrifolia (Heliozelidae, Gracilariidae). *J. Lepid. Soc.* **25,** 194–211(1971).
391. Opler, P. A. (1998). *A field guide to western butterflies*. Houghton Mifflin Harcourt.
392. Opler, P. A. & Davis, D. R. *The Leaf Mining Moths of the Genus Cameraria Associated with Fagaceae in California (Lepidoptera: Gracillariidae)* (Smithsonian Institution Press., 1981).
393. Ostry, M. E. & Henderson, F. L. *Populus: a bibliography of world literature, 1975-1988* (National Agricultural Library, 1990).
394. Otto, H. *Butterflies of the Kruger National Park and surrounds* (Penguin Random House South Africa, 2014).
395. Pacific Northwest Moths. Species Fact Sheets <https://pnwmoths.biol.wwu.edu> (2024).
396. Packham, C. *Wildlife of the World* (Dorling Kindersley Ltd, 2015).
397. Paine, T. D. & Lieutier, F. *Insects and diseases of Mediterranean forest systems*. (Springer, 2016).
398. Pankoke, H., Gehring, R. & Müller, C. Impact of the dual defence system of Plantago lanceolata (Plantaginaceae) on performance, nutrient utilisation and feeding choice behaviour of Amata mogadorensis larvae (Lepidoptera, Erebidae). *J. Insect Physiol.* **82**, 99–108 (2015).
399. Park, K.-T., Heo, U.-H. & Byun, B.-K. Two new species and three unrecorded species of Gelechiidae (Lepidoptera: Gelechioidea) from Korea, with biological data including larval host plants. *J. Asia-Pac. Biodivers.* **13**, 605–612 (2020).
400. Park, K. T., Heo, U. H., Kim, D. S. & Byun, B. K. A review of larval host plants with some biological notes of the family Gelechiidae (Lepidoptera) in Korea. *J. Asia-Pac. Biodivers.* **15,** 1–8 (2022).
401. Parra, L. E. & Cerda, L. A. Presencia de Proeulia chrysopteris (Butler)(Lepidoptera: Tortricidae) sobre Pinus radiata D. Don. *Bosque* **12**, 61–63 (1991).
402. Pascual, J. A. Biologia de la Procesionaria del roble (Thaumetopoea processionea L.)(Lep. Thaumetopoeidae) en el centro-oeste de la Peninsula Iberica. *Boletin de Sanidad Vegetal Plagas* **14**, 383–404 (1988).
403. Pasek, J. E. & Ellen Dix, M. Life history of a ponderosa pine coneworm, Dioryctria auranticella (Lepidoptera: Pyralidae). *J. Econ. Entomol.* **82**, 879–885 (1989).
404. Patrick, B. & Dugdale, J. Australian lucerne leafroller, Merophyas divulsana (Lepidoptera: Tortricidae), in New Zealand. *N. Z. J. Zool.* **21**, 305–308 (1994).
405. Patrick, B. & Kleinpaste, R. Moths from a conifer nest in Christchurch. *The Wētā* **47**, 39–46 (2014).
406. Patrick, C. R. Observations on the life history of *Periploca mimula* in Tennessee (Lepidoptera: Walshiidae*). Journal of the Georgia Entomological Society,* **10,** 259-261 (1975).
407. Peigler, R. S. *A revision of the Indo-Australian genus Attacus (Lepidoptera: Saturniidae)* (Texas A&M University, 1983).
408. Peigler, R. S. Life history of Olceclostera seraphica (Lepidoptera: Bombycidae, Apatelodinae). *Nachrichten des Entomologischen Vereins Apollo, Frankfurt (Neue Folge)* **15**, 245–252 (1994).
409. Pellmyr, O., Balcázar‐Lara, M., Althoff, D. M., Segraves, K. A. & Leebens‐Mack, J. Phylogeny and life history evolution of Prodoxus yucca moths (Lepidoptera: Prodoxidae). *Syst. Entomol.* **31**, 1–20 (2006).
410. Pemberton, C. E. Highlights in the history of entomology in Hawaii 1778–1963. *Pacific Insects* **6**, 689–729 (1964).
411. Pereira, F. F. *et al.* Aspectos biológicos de Halysidota pearsoni (Lepidoptera: Arctiidae) com folhas de amoreira. *Revista Árvore* **31**, 157–161 (2007).
412. Pfannenstiel, R. S., Unruh, T. R. & Brunner, J. F. Overwintering hosts for the exotic leafroller parasitoid, Colpoclypeus florus: Implications for habitat manipulation to augment biological control of leafrollers in pome fruits. *J. Insect Sci.* **10**, 75 (2010).
413. Picker, M. *Field guide to insects of South Africa* (Penguin Random House South Africa, 2012).
414. Pitkin, B., Ellis, W., Plant, C. & Edmunds, R. The leaf and stem mines of British flies and other insects (Coleptera, Diptera, Hymenoptera and Lepidoptera) <http://www.ukflymines.co.uk> (2019).
415. Pittaway, A. R. Sphingidae of the Western Palaearctic (including Europe, North Africa, the Middle East, western Siberia and western Central Asia) <https://tpittaway.tripod.com/sphinx/list.htm> (2024).
416. EFSA Panel on Plant Health (PLH). Pest categorisation of non‐EU Acleris spp. [*https://doi.org/10.2903/j.efsa.2019.5856*](https://doi.org/10.2903/j.efsa.2019.5856) (2019).
417. EFSA Panel on Plant Health (PLH). Pest categorisation of *Spodoptera eridania* [*https://doi.org/10.2903/j.efsa.2020.5932*](https://doi.org/10.2903/j.efsa.2020.5932) (2020).
418. EFSA Panel on Plant Health (PLH). Pest categorisation of *Amyelois transitella* [*https://doi.org/10.2903/j.efsa.2021.6666*](https://doi.org/10.2903/j.efsa.2021.6666) (2021).
419. Pogue, M. *The Noctuinae (Lepidoptera: Noctuidae) of Great Smoky Mountains National Park, USA* (Magnolia Press, 2006).
420. Pogue, M*. A review of selected species of Lymantria Hübner (1819)(Lepidoptera: Noctuidae: Lymantriinae) from subtropical and temperate regions of Asia, including the descriptions of three new species, some potentially invasive to North America* (US Forest service, 2007).
421. Poltavsky, A. N. *et al.* The Pyraloidea (Lepidoptera) fauna of the woody savannah belt in Mali, West Africa. *Zootaxa* **4457**, 39 (2018).
422. Ponomarenko, M. G. Catalogue of the subfamily Dichomeridinae (Lepidoptera, Gelechiidae) of the Asia. *Far East. Entomol.* **50,** 1–67 (1997).
423. Porter, A. H. (1986). Life history *of Nemoria glaucomarginaria*(Barnes & McDunnough) and larval taxonomy of the tribe Nemoriini(Geometridae: Geometrinae). *Journal of the Lepidopterists Society*, *40*(4), 304–314.
424. Porter, J. *Colour Identification Guide to Caterpillars of the British Isles:(Macrolepidoptera)*. (Apollo Books, 2010).
425. Powel, J. A. & Brown, J. W. *The Moths of North America. Fascicle 8.1, Tortricoidea, Tortricidae (Part), Sparganothini and* Atterini (Wedge Entomological Research Foundation, 2012).
426. Powell, J. A. A review of Griselda, with description of a related new genus and two new species (Lepidoptera: Tortricidae). *J.* *Pan-Pac. Entomol.* **40**, 85–97 (1964).
427. Powell, J. A. *A systematic monograph of New World ethmiid moths (Lepidoptera: Gelechioidea)*. (Smithsonian Institution Press, 1973).
428. Powell, J. A. (1980). *Nomenclature of nearctic conifer-feeding Choristoneura (Lepidoptera: Tortricidae): Historical review and present status*. vol. 100. Gen. Tech. Rep. PNW-GTR-100 (U.S. Department of Agriculture, Forest Service, 1980).
429. Powell, J. A. A synopsis of Decodes (Lepidoptera: Tortricidae), with descriptions of new species and a related new genus in Mexico. *Pacific Insects* **22**, 78–114 (1981).
430. Powell, J. A. *Biosystematic studies of conifer-feeding Choristoneura (Lepidoptera: Tortricidae) in the western United States*. vol. 115. (Univ of California Press, 1995).
431. Powell, J. A. & Brown, J. W. *The Moths of North America north of Mexico Fascicle 8.1. Tortricidae (Part) Sparganothini and Atteriini* (Wedge Entomological Research Foundation, 2012).
432. Powell, J. A. & Opler, P. A. Larval host plant records of Asteraceae root-feeding Eucosmini in California and adjacent states (Tortricidae). *J. Lepid. Soc.* **60**, 189 (2006).
433. Powell, J. A. & Opler, P. A. *Moths of western north america* (University of California Press, 2009).
434. Praciak, A. *The CABI encyclopedia of forest trees* (CABI, 2013).
435. Predovnik, Z. Clearwing moths (Lepidoptera: Sesiidae) new to the fauna of Slovenia. *Slovenian Entomological Society* **13,** 93–102 (2005).
436. Prout, L. B. The Palaearctic Geometrae. *In: The Macrolepidoptera of the World.* Vol. 4. (Ed. A. Seitz). (Verlag A Kernen: Stuttgart, Germany, 1912).
437. Pyle, R. M. & LaBar, C. C. *Butterflies of the Pacific Northwest* (Timber Press, 2018).
438. Raghu, S., Morin, L. & Pratt, P. *Prioritizing weed targets for biological control in the western USA*. *Health & Biosecurity.* Clayton South Victoria, Australia: Commonwealth Scientific and Industrial Research Organization 96819 (2018).
439. Rai, M. K., Varma, A. & Rajak, R. C. Integrated management of plant resources. (2000).
440. Ramawat, K. G. *Biotechnology of medicinal plants: vitalizer and therapeutic*. (CRC Press, 2004).
441. Ramírez de López, M. B. & Gómez López, V. A. Biología de Spodoptera eridania (Cramer, 1782)(Lepidoptera; Noctuidae) en dieta natural y artificial, en condiciones de laboratorio. *Investigación Agraria* **12**, 17–21 (2010).
442. James, D. G. *The book of caterpillars: a life-size guide to six hundred species from around the world* (University of Chicago Press, 2017).
443. Ravoet, J. & Mol, A. W. M. The sawfly Arge dimidiata in Belgium and the Netherlands (Hymenoptera: Argidae). *Bulletin de La Société Royale Belge d’Entomologie*, **154,** 138–142 (2018).
444. Red Europea a gran escala de seguimiento de los bosques (NIVEL I) Acción L2-23(ES)/FUTMON Islas Canarias. 2009. Ministerio de Medio Ambiente y Medio Rural y Marítimo. Gobierno de España <https://www.miteco.gob.es/content/dam/miteco/es/biodiversidad/temas/inventarios-nacionales/islas_canarias_2009_tcm30-155562.pdf> (2009).
445. Rennwald, E. & Rodeland, J. Lepiforum eV. Identification of Butterflies and their Preimaginal Stages v.12. <https://lepiforum.org/> (2022).
446. OMNR. (1998). *A silvicultural guide for the tolerant hardwood forest in Ontario. v1.1*. Peterborough: Ontario Ministry of Natural Resources. Queen´s Printer for Ontario. Toronto. <https://dr6j45jk9xcmk.cloudfront.net/documents/2820/siv-guide-tolerant-hardwood.pdf> (1998).
447. Richards, R. A. *The pelagic dictionary of natural history of the British Isles* (Pelagic Publishing, 2020).
448. Rings, R. W. Contributions to the bionomics of the green fruitworms: the life history of Lithophane laticinerea. *J. Econ. Entomol.* **62**, 1388–1393 (1969).
449. Ríos de Saluso, M. L. A. Artrópodos asociados al “Caraguatá,” *Eryngium paniculatum* (Apiaceae). *Revista Científica Agropecuaria*, **1,** 35–37 (1997).
450. Riotte, J. C. E. The moths of America north of Mexico, including Greenland: Fascicle 22.2, Noctuoidea (in part): Lymantriidae, by D. C. Ferguson [Review]. *The Canadian Field-Naturalist* **93,** 203–205 (1979).
451. Robinson, G. S., Ackery, P. R., Kitching, I., Beccaloni, G. W. & Hernández, L. M. HOSTS - a Database of the World’s Lepidopteran Hostplants. Natural History Museum [*https://doi.org/10.5519/havt50xw*](https://doi.org/10.5519/havt50xw) (2023).
452. Robinson, G. S., Ackery, P. R., Kitching, I. J., Beccaloni, G. W. & Hernández, L. M. *Hostplants of the moth and butterfly caterpillars of the Oriental Region* (The Natural History Museum, London, 2001).
453. Rocchini, L. A., Lindgren, B. S. & Bennett, R. G. Douglas-fir pitch moth, Synanthedon novaroensis (Lepidoptera: Sesiidae) in North-Central British Columbia: Flight period and the effect of trap type and pheromone dosage on trap catches. *Environ.l Entomol.* **32**, 208–213 (2003).
454. Rodríguez-Ortega, L. T. Primer registro de *Leucanella viridescens* Walker, 1855 (Lepidoptera: Saturniidae: Hemileucinae) en el cultivo de morera (Morus alba Linn.). *Agro Productividad*, **11** (2018).
455. Ross, D. A. & Evans, D. Annotated list of forest insects of British Columbia Part IV-Hydriomena spp.(Geometridae). *J. Entomol. Soc. of B. C.* **52**, 38–39 (1956).
456. Roy, S., Das, S., Handique, G., Mukhopadhyay, A. & Muraleedharan, N. Ecology and management of the black inch worm, Hyposidra talaca Walker (Geometridae: Lepidoptera) infesting Camellia sinensis (Theaceae): A review. *J. Integr. Agric.* **16**, 2115–2127 (2017).
457. Roychoudhury, N. & Mishra, R. K. Deodar looper, Ectropis deodarae and its control measures. *Tropical Forest Research Institute* **8,** 14-16 (2021).
458. Royer, T. A. & Knodel, J. J. Sunflower moth (Lepidoptera: Pyralidae) biology, ecology, and management. *J. Integr. Pest Manag.* **10,** 25 (2019.
459. Rutherford, M. G. Notes on the Life History of *Citheronia hamifera* Rothschild, 1907 (Lepidoptera, Saturniidae). *Living World, Journal of the Trinidad and Tobago Field Naturalists’ Club*, 36 (2019).
460. Saarenmaa, H. Taxonomy and ecology of Cosmotriche lunigera (Lepidoptera, Lasiocampidae) in Finland, with a description of a northern subspecies. *Not. Entomol.* **623**, 97–109 (1982).
461. Sabourin, M., Priest, R. J. & Miller, W. E. Nearctic Acleris: resurrection of A. stadiana and a revised identity for A. semiannula (Lepidoptera: Tortricidae). *The Great Lakes Entomologist* **30**, 1 (1997).
462. Sakagami, K. Review of the species of Sorolopha Lower (Lepidoptera: Tortricidae) in Japan, with descriptions of three new species. *Zootaxa* **5278**, 131–142 (2023).
463. Solomon, J. D. *Guide to insect borers in North American broadleaf trees and shrubs* (US Department of Agriculture, Forest Service, 1995).
464. Sangha, K. S. Evaluation of management tools for the control of poplar leaf defoliators (Lepidoptera: notodontidae) in northwestern India. *J. For. Res.* **22**, 77–82 (2011).
465. Saunders, J. L. *Plagas invertebradas de cultivos anuales alimenticios en América Central*. vol. 52 (Catie, 1998).
466. Savela, M. Lepidoptera and some other life forms. FUNET <https://ftp.funet.fi/index/Tree_of_life/insecta/> (2021).
467. Scalercio, S. & Malaisse, F. Between species and ethnospecies: edible Psychidae in Tropical Africa. *Entomologie Faunistique* **62**, (2009).
468. Schiff, N. M. *et al.* Siricidae (Hymenoptera: Symphyta: Siricoidea) of the Western Hemisphere. *Can. J. Arthropod Identif.* **21,** 1–305 (2012).
469. Schintlmeister, A. *Notodontidae*. vol. 1. (Brill, 2013).
470. Schmidt, B. C., Wagner, D. L., Zacharczenko, B. v, Zahiri, R. & Anweiler, G. G. Polyphyly of Lichen-cryptic Dagger Moths: synonymy of Agriopodes Hampson and description of a new basal acronictine genus, Chloronycta, gen. n.(Lepidoptera, Noctuidae). *ZooKeys* **115** (2014).
471. Schmutterer, H. Beobachtungen an Schädlingen von Azadirachta indica (Niembaum) und von verschiedenen Melia‐Arten. *J. Appl. Entomol.* **109**, 390–400 (1990).
472. Schweitzer, D. F. *A revision of the genus Metaxaglaea (Lepidoptera: Noctuidae, Cuculliinae) with descriptions of two new species* (Postilla No. 178, Peaboby Museum of Natural History, New Haven, 1979a).
473. Schweitzer, D. F. Predatory behavior in Lithophane querquera and other spring caterpillars. *J. Lepid. Soc.* ***33*,** 129–134 (1979b).
474. Schweitzer, D. F., Minno, M. C. & Wagner, D. L. Rare, declining, and poorly known butterflies and moths (Lepidoptera) of forests and woodlands in the eastern United States (US Forest Service. Forest Health Assessment and Applied Sciences Team, 2011).
475. Scott, J. A. *The butterflies of North America: a natural history and field guide* (Stanford University Press, 1992).
476. Seitz, A. *The Macrolepidoptera of the World: A Systematic Description of the Known Macrolepidoptera Edited with the Collaboration of Well-known Specialists*. vol. 1 (A. Kernen, 1906).
477. Senckenberg. Insekten Sachsen. Occurrence dataset <https://doi.org/10.15468/ops3q2> (2024).
478. *Forest insect and disease conditions in the United States* (U.S. Deptartment of Agriculture, Forest Service, 1972).
479. Sganga, J. V. & Fontanarrosa, M. S. Contribution to the knowledge of the preimaginal stages of the genus Smicridea McLachlan in South America (Trichoptera: Hydropsychidae: Smicrideinae). *Zootaxa* **1258**, 1–15 (2006).
480. Shaw, R. H., Cock, M. J. W. & Evans, H. C. The natural enemies of privets (Ligustrum: Oleaceae): a literature review, with particular reference to biological control. *CABI Reviews* 1–24 (2018).
481. Shea, P. J. Interactions among phytophagous insect species colonizing cones of white fir (Abies concolor). *Oecologia* **81**, 104–110 (1989).
482. Shepard, J. & Guppy, C. *Butterflies of British Columbia: including western Alberta, southern Yukon, the Alaska panhandle, Washington, northern Oregon, northern Idaho, and northwestern Montana* (UBC Press, 2001).
483. Shin, S.-B. & Byun, B.-K. Taxonomic review of the genus Zeiraphera Treitschke (Lepidoptera: Tortricidae) in Korea, with description of a new species. *J. Asia-Pac. Biodivers.* **9**, 22–28 (2016).
484. Shin, Y.-M., Lee, B.-W. & Byun, B.-K. Taxonomic review of the genus Caloptilia Hübner (Lepidoptera: Gracillariidae) in Korea. *J. Asia-Pac. Entomol.* **18**, 83–92 (2015).
485. Shin, Y.-M., Nam, J.-W., Kim, D.-K., Byun, B.-K. & Kim, I.-K. Two lepidopteran pests and damage on the cones of Abies koreana (Pinaceae) in Jeju Island, Korea. *J. Asia-Pac. Biodivers.* **11**, 80–86 (2018).
486. Shiraiwa, K., Cong, Q. & Grishin, N. v. A new Heraclides swallowtail (Lepidoptera, Papilionidae) from North America is recognized by the pattern on its neck. *ZooKeys* **85** (2014).
487. Sihvonen, P. & Skou, P. *Ennominae i*. vol. 5 (Brill, 2015).
488. Silva, E. B. & Mexia, A. The pest complex Cryptoblabes gnidiella (Millière)(Lepidoptera: Pyralidae) and Planococcus citri (Risso)(Homoptera: Pseudococcidae) on sweet orange groves (Citrus sinensis (L.) Osbeck) in Portugal: interspecific association. *Bol. San. Veg. Plagas* **25,** 89–98 (1999).
489. Simmons, P., Reed, W. D. & McGregor, E. A. *Fig insects in California* (US Department of Agriculture, 1931).
490. Singh, A. *Insect Pests of Western Himalayan Oaks in Uttarakhand* (Forest Research Institute (ICFRE), 2022).
491. Singh, A. P., Bahuguna, K. & Ramola, G. C. New host records of polyphagous Lepidoptera on Ban Oak Quercus leucotrichophora A. Camus (Fabaceae) in the Garhwal Himalaya, India. *J. Threat. Taxa* **11**, 13579–13591 (2019).
492. Singh, I. K. & Singh, A. *Plant-pest interactions: from molecular mechanisms to: chemical ecology* (Springer, 2021).
493. Singh Rathore, M. P. *Insect pests in agroforestry* (International Centre for Research in Agroforestry, 1995).
494. Skou, P. *The Geometroid Moths of North Europe (Lepidoptera: Drepanidae and Geometridae)*. vol. 6. (Brill Archive, 1986).
495. Skou, P. & Sihvonen, P. M. *The* *Geometrid Moths of Europe (Lepidoptera: Ennominae I).* vol. 5. (Brill Archive, 2015).
496. Slootmaekers, D., Snyers, C. & Wullaert, S. Ephestia welseriella and Delplanqueia inscriptella (Lepidoptera: Pyralidae, Phycitinae), new to the Belgian fauna. *Phegea* **45**, 91–93 (2018).
497. SLU Artdatabanken. The species portal <https://www.slu.se/> (2024).
498. Sohn, J. C., Ronkay, L. & Choi, S. W. First report of five noctuid species (Lepidoptera: Noctuidae) from Korea. *J. Asia-Pac. Entomol.* **8**, 147–152 (2005).
499. Sohn, J.-C. & Choi, S.-W. Two species of Olethreutinae (Lepidoptera: Tortricidae) new to Korea. *J. Species Res.* **9**, 167–169 (2020).
500. Sohn, J.-C., Park, K.-T., kyun Lee, S. & Cho, S. A taxonomic review of the genus Batrachedra (Lepidoptera: Gelechioidea: Batrachedridae) in Korea. *J. Asia-Pac. Entomol.* **12**, 101–105 (2009).
501. Solis, M. A. Pyraloidea and their known hosts (Insecta: Lepidoptera) of Plummers Island, Maryland. *Biol. Soc. Wash. Bull.* **15**, 88–106 (2008).
502. Sourakov, A. & Chadd, R. W. *The lives of moths: A natural history of our planet’s moth life*. vol. 1. (Princeton University Press, 2022).
503. Sourakov, A. & Stubina, M. Scientific Note: Functional morphology of masquerading larva of Ceratonyx satanaria with notes on horned spanworm, Nematocampa resistaria (Geometridae: Ennominae). *Trop. Lepid. Res.* **22,** 53–59 (2012).
504. South, R. The Moths of the British Isles, (First Series), Frederick Warne & Co. Ltd., London & NY. Online <https://www.gutenberg.org/files/41782/41782-h/41782-h.htm> (1907).
505. Souza, J. S. I., Peixoto, A. M. & de Toledo, F. F. *Enciclopédia agrícola brasileira: AB*. vol. 1. (Edusp, 1995).
506. Specht, A., Formentini, A. C. & Corseuil, E. Biologia de Automeris illustris (Walker)(Lepidoptera, Saturniidae, Hemileucinae). *Ver. Bras. Zool.* **23**, 537–546 (2006).
507. Specht, A., Fronza, E., Colombi, E. E., Formentini, A. C. & Corseuil, E. Aspects of the biology and morphology of Dirphia dolosa Bouvier, 1929 (Lepidoptera: Saturniidae: Hemileucinae). *Biota Neotrop.* **12**, 21–28 (2012).
508. Spencer, L. A. & Simons, D. R. *Arkansas butterflies and moths* (University of Arkansas Press, 2006).
509. St Laurent, R. A. *et al.* Phylogenetic systematics, diversification, and biogeography of Cerurinae (Lepidoptera: Notodontidae) and a description of a new genus. *Insect Syst. Divers.* **7**, 3 (2023).
510. Staude, H. *et al.* Bombycoidea: Saturniidae: Bunaeinae, Saturninae; Sphingidae: Macroglosiinae, Sphinginae. *Metamorphosis* **31**, 34–53 (2022).
511. Staude, H., Picker, M. & Griffiths, C. *Southern African moths & their caterpillars* (Penguin Random House South Africa, 2023).
512. Staude, H. S. *et al.* An overview of Lepidoptera-host-parasitoid associations for southern Africa, including an illustrated report on 2 370 African Lepidoptera-host and 119 parasitoid-Lepidoptera associations. *Metamorphosis* **31**, 1–380 (2020).
513. Stevens, R. E. *Lepidoptera associated with western spruce budworm* (US Department of Agriculture, Forest Service, Cooperative State Research Service, 1984).
514. Stevens, R. E. Life history and habits of Exoteleia anomala Hodges, a ponderosa pine needle miner in the southwestern United States (Gelechiidae). *J. Lepid. Soc*. **40,** 23–26 (1986).
515. Stevens, R. E., Brewer, J. W. & Jennings, D. T. Life history and habits of Coleotechnites edulicola (Gelechiidae), a pinyon needle miner in the southwest. *J. Lepid. Soc*. **32,** 123–129 (1986). (1978).
516. Still, G. N. & Wong, H. R. Life history and habits of a leaf miner, Cameraria macrocarpae, on bur oak in Manitoba (Lepidoptera: Gracillariidae). *Can. Entomol.* **105**, 239–244 (1973).
517. Stockley, C. H. *Some East African Hawk Moths*. *XXII*. Biodiversity library <https://www.biodiversitylibrary.org/content/part/EANHS/XXII_No.1_93__18_1953_Stockley.pdf> (1953).
518. Sugimoto, M. & Saigusa, T. The systematic position, morphology and bionomics of Acanthopsyche (Eumetisa) taiwana Sonan, 1935, newly recorded from the Ryukyus, Japan (Lepidoptera: Psychidae). *Entomol. Sci.* **4,** 407–430 (2001).
519. Sugiura, S. & Yamazaki, K. Outbreak of Ivela auripes (Lepidoptera, Lymantridae) and its arboreal predator Dendroxena sexcarinata (Coleoptera, Silphidae). *Lepid. Sci.* **58**, 40–42 (2007).
520. Šumpich, J. & Skyva, J. Dichomeris barbella (Denis et Schiffermüller, 1775)(Lepidoptera: Gelechiidae) v Doupovských horách–potvrzený výskyt v České republice po 150 letech. *Klapalekiana,* **52,** 67–77 (2016).
521. Swiecki, T. J. & Bernhardt, E. A. *A field guide to insects and diseases of California oaks* (US Department of Agriculture, 2006).
522. Taft, W. H., Smitley, D. & Snow, J. W. *A guide to the clearwing borers (Sesiidae) of the north central United States* (USDA North Central Regional Publication, 1991).
523. *Taiwan Encyclopedia of Life (TaiEOL*) <https://taieol.tw> (2024).
524. Tatum, J. B. Butterflies and Moths of the Southern Vancouver Island <http://vicnhs.bc.ca/lepsvi/> (2024).
525. Terada, T. Two new species of the genus Stathmopoda (Lepidoptera: Stathmopodidae) closely related to S. opticaspis from Japan. *Lepid. Sci.* **64**, 140–149 (2013).
526. Tewari, D. N. *A monograph on gamari (Gmelina arborea Roxb.)* (International Book Distributions, 1995).
527. The Missouri Department of Conservation. “*Field Guide Magdalen Underwing”* <https://mdc.mo.gov/discover-nature/field-guide/magdalen-underwing> (2024).
528. Tikader, A., Vijayan, K. & Saratchandra, B. Cricula trifenestrata (Helfer)(Lepidoptera: Saturniidae)-A silk producing wild insect in India. *Trop. Lepid. Res.* **24,** 22–29 (2014).
529. Titus, F. A. *Pests that Changed the Forests of Fundy National Park*. Issue MX–165 (Canadian Forestry Service, 1987)
530. TNAU agritech portal. Crop protection. <https://agritech.tnau.ac.in/crop_protection/crop_prot_crop_insect_pest.html> (2015).
531. Trematerra, P. & Brown, J. W. Argentine Argyrotaenia (Lepidoptera: Tortricidae): Synopsis and descriptions of two new species. *Zootaxa* **574**, 1–12 (2004).
532. Triggiani, O. Osservazioni sulla bioetologia della Dioryctria pineae (Stgr.)(Lepidoptera: Phycitidae) e sul suo parassitoide Elachertus geniculatus (Ratz.)(Hymenoptera: Chalcidoidea). *Entomologica* **21**, 141–153 (1986).
533. Trimen, R. & Bowker, J. H. *South-African butterflies: a monograph of the extra-tropical species*. vol. 3. (London Trübner, 1889).
534. Tolman, T. *Collins butterfly guide* (HarperColins UK, 2008).
535. Tuskes, P. M., Collins, M. M. & Tuttle, J. *The wild silk moths of North America: a natural history of the Saturniidae of the United States and Canada* (Cornell University Press, 2019).
536. United States Congress House. Committee on Appropriations. Subcommittee on Agriculture Food and Drug Administration and Related Agencies. *Agriculture, Rural Development, Food and Drug Administration, and Related Agencies Appropriations for 2004: Hearings Before a Subcommittee of the Committee on Appropriations, House of Representatives, One Hundred Eighth Congress, First Session* <https://books.google.es/books?id=iVlRAQAAMAAJ> (U.S. Government Printing Office. , 2011).
537. University of Massachusetts Amherst. UMass Extension Landscape, Nursey and Urban Forestry Program <https://ag.umass.edu/landscape/publications-resources/insect-mite-guide> (2024).
538. USDA Forest Service. Insects & Diseases <https://www.fs.usda.gov/detailfull/r8/forest-grasslandhealth/insects-diseases/> (2024).
539. University of Alberta E.H. Strickland Entomological Museum*.* Collection datasets <https://www.ualberta.ca/en/museums/museum-collections/eh-strickland-entomological-museum.html> (2024)
540. van den Berg, M. A. Pouoogmotte (Lepidoptera: Saturniidae) van die Nasionale Krugerwildtuin. *Koedoe* **17**, 159–172 (1974).
541. van Driesche, R., LaForest, J. H., Bargeron, C. T., Reardon, R. C. & Herlihy, M. *Forest pest insects in North America: a photographic guide* (USDA Forest Service Morgantown, West Virginia, 2013).
542. van Duzee, E. P. *The Pan-Pacific Entomologist*. vol. 1. (Pacific Coast Entomological Society., 1924).
543. van Nieukerken, E. A taxonomic revision of the western Palaearctic species of the subgenera Zimmermannia Hering and Ectoedemia Busck s. str.(Lepidoptera, Nepticulidae), with notes on their phylogeny. *Tijdschrift voor Entomologie* **28**, 1–98 (1985).
544. Varatharajan, R., Sudhakar, S., Reeta, L., Singh, N. I. & Mathavan, S. Occurrence of reovirus in oak tasar silkworm Antheraea proylei. *Curr. Sci.* **75,** 724–726 (1998).
545. Vargas, H. A. Lycaenid caterpillars (Lepidoptera, Lycaenidae) eating flowers of Dalea pennellii var. chilensis (Fabaceae) in the northern Chilean Andes. *Rev. Bras. Entomol.* **58**, 309–312 (2014).
546. Venette, R. C., Davis, E. E., Heisler, H. & Larson, M. *Mini risk assessment, silver Y moth, Autographa gamma (L.)[Lepidoptera: Noctuidae].* USDA APHIS PPQ (U.S. Department of Agriculture, 2003).
547. Vikberg, V. & Viitasaari, M. Trichiosoma nanae sp. n., a monophag on Betula nana from Finland (Hymenoptera, Cimbicidae). *Entomol. Fenn.* **2**, 67–77 (1991).
548. Villanueva, R. T., Rodrigues, J. C. v & Childers, C. C. Larval Cryptothelea gloverii (Lepidoptera: Psycidae), an arthropod predator and herbivore on Florida citrus. *Exp. Appl. Acarol.* **36**, 83–92 (2005).
549. Virginia Department of Conservation and Recreation - Division of Natural Heritage and Virginia Department Wildlife Resources. *Atlas of Rare Butterflies, Skippers, Moths, Dragonflies, and Damselflies of Virginia* <https://www.vararespecies.org/> (2013).
550. Wagner, D. L. Taxonomic status of Korscheltellus Börner in North America (Lepidoptera: Hepialidae). *J. N. Y. Entomol. Soc.* **96,** 345–354 (1988).
551. Wagner, D. L. *Geometroid caterpillars of northeastern and Appalachian forests* (US Department of Agriculture, Forest Service, 2001).
552. Wagner, D. L. *Caterpillars of eastern North America: a guide to identification and natural history* (Princeton University Press, 2010).
553. Wagner, D. L., Loose, J. L., Fitzgerald, T. D., de Benedictis, J. A. & Davis, D. R. A hidden past: the hypermetamorphic development of Marmara arbutiella (Lepidoptera: Gracillariidae). *Ann. Entomol. Soc. Am.* **93**, 59–64 (2000).
554. Wagner, D. L. & McCabe, T. L. A new Zanclognatha from eastern North America and a preliminary key to the larvae of the genus (Lepidoptera, Erebidae, Herminiinae). *ZooKeys* **89** (2011).
555. Wagner, M. R., Cobbinah, J. R. & Bosu, P. P. *Forest entomology in West Tropical Africa: forest insects of Ghana* (Springer Science & Business Media, 2008).
556. Wagner, W. Lepidoptera and their ecology <http://pyrgus.de/> (2024).
557. Wali-ur-Rahman, R. & Chaudhry, M. I. Observations on outbreak and biology of oak defoliator, Gazalina chrysolopha Koll. *Pak. J. For.* 134-137 (1992).
558. Wang, Z. F. & Wang, P. Y. The bionomics and control of a pyralid insect pest (Euzophera batangensis) of China fir in China. *Econ. Botan.* **42,** 376–406 (1988).
559. Ward, K. E., Ramaswamy, S. B. & Nebeker, T. E. Feeding preferences and their modification in early and late instar larvae of the bagworm, Thyridopteryx ephemeraeformis (Lepidoptera: Psychidae). *J. Insect Behav.* **3**, 785–795 (1990).
560. Watt, G. *The Pests and Blights of the Tea Plant: Being a Report of Investigations Conducted in Assam and to Some Extent Also in Kangra* (Office of the superintendent, Government printing, India, 1898).
561. Weber, L. M. & Weber, J. *Native Host Plants for Texas Moths: A Field Guide* (Texas A&M University Press, 2022).
562. Webster, R. P. & Thomas, A. W. A new species of Lithophane (Lepidoptera: Noctuidae: Cuculliinae) from northeastern North America. *J. Lepid. Soc.* **53,** 55–59 (1999).
563. Weller, S. J. Litodonta hydromeli Harvey (Notodontidae): description of life stages. *J. Lepid. Soc.* **41**, 187–194 (1987).
564. Wheeler, A. G. & Hoebeke, E. R. The insect fauna of ninebark, Physocarpus opulifolius (Rosaceae). *Proc. Entomol. Soc. Wash.* **87**, 356–370 (1985).
565. Wheeler, J. Norfolk Online Lepidoptera Archive - NOLA <https://www.suffolkmoths.co.uk> (2024).
566. Whitebread, S., Mello, M., Murray, T., & Zimberlin, D. MassMoths <https://massmoths.org/> (2024).
567. Whitehouse, C. M. Seasonal phenology and reproductive behaviour of Dioryctria species Zeller (Lepidoptera: Pyralidae) in British Columbian seed orchards (2011).
568. Whossler, E. The larva and pupa of Lytrosis permagnaria pack.(Geometridae). *J. Lepid. Soc.* **57**, 107–112 (2003).
569. Wilkinson, C. & Scoble, M. J. The Nepticulidae (Lepidoptera) of Canada. *Mem. Entomol.Soc. Can.* **111**, 1–129 (1979).
570. Will, K., Gross, J., Rubinoff, D. & Powell, J. A. *Field guide to California insects* (University of California Press, 2020).
571. Willson, K. C. & Clifford, M. N. *Tea: cultivation to consumption* (Springer Science & Business Media, 2012).
572. Wilson, L. F. & Heaton, G. C. Notes on the life cycle of Nemoria rubrifrontaria (Lepidoptera: Geometridae). *Gt. Lakes Entomol.* **7**, 13 (2017).
573. Winkels, B. “Erynnis persius” (On-line), Animal Diversity Web <https://animaldiversity.org/accounts/Erynnis_persius/> (2014).
574. Wolfe, K. L. Three new species of Paradirphia (Saturniidae: Hemileucinae) from Mexico and Central America with. *J. Res. Lepid.* **27**, 197–212 (1988).
575. Wolfe, K. L. & Balcázar-Lara, M. A. An illustrated description immature stages of Lonomia electra (Druce, 1886)(Lepidoptera: Saturniidae, Hemileucinae). *Nachrichten des Entomologischen Vereins Apollo* **15**, 383–392 (1994).
576. Wolfe, K. L., Lemaire, C., Amarillo S, A. & Conlan, C. A. A contribution to the systematics of the Copaxa semioculata species-group (Saturniidae), with notes on the early stages, and a description of Copaxa lunula*,* new species. *J. Lepid. Soc.* **57,** 54–61 (2003).
577. Wolfe, K. L. & Peigler, R. S. Life history of Anisota dissimilis (Lepidoptera: Saturniidae: Ceratocampinae). *Trop. Lepid. Res.* 4, 143–145 (1993).
578. Wolfgang A, N. & Min, W. First record of the saturniid genus Lemaireia Nässig & Holloway, 1987 from Hainan island (PR China) with the description of a new species (Lepidoptera: Saturniidae). *Nachrichten des Entomologischen Vereins Apollo* **27**, 23–25 (2006).
579. Wood, C. S., van Sickle, G. A. & Shore, T. L. *Forest insect and disease conditions British Columbia & Yukon* (Canadian Forestry Service, Pacific Forestry Centre, 1986).
580. Woodhall, S. *Field guide to butterflies of South Africa* (Bloomsbury Publishing, 2020).
581. Worrall, J. & Geils, B. Dwarf mistletoes. The Plant Health Instructor <http://www.apsnet.org/edcenter/intropp/lessons/miscellaneous/Pages/Dwarfmistletoes.aspx> (2006).
582. Wylie, F. R. & Speight, M. R. *Insect pests in tropical forestry* (CABI, 2012).
583. Yamazaki, K. & Sugiura, S. Stem‐galling moths provide cetoniine beetles with feeding sites via sap exudation of invasive alien plants. *Entomol. Sci.* **19**, 142–146 (2016).
584. Yano, K. On the larva and pupa of Commatarcha palaeosema Meyrick, with its biological notes (Lepidoptera, Carposinidae). *Kontyû* **27**, 214–217 (1959).
585. Yen, S. H. Y. S. & Jan, J. L. Notes on the life history of *Sibataniozephyrus kuafui* Hsu & Lin, 1994 (Lepidoptera: Lycaenidae). *Chinese J. Entomol.* **15,** 161–169 (1995).
586. Ylla, J., Peigler, R. S. & Kawahara, A. Y. Cladistic analysis of moon moths using morphology, molecules, and behaviour: Actias Leach, 1815; Argema Wallengren, 1858; Graellsia Grote, 1896 (Lepidoptera: Saturniidae). *SHIL. Rev. Lepidopterol.* **33**, 299–317 (2005).
587. Yoshida, K. & Murakami, M. Larval morphology and feeding behavior in Notodontidae (Lepidoptera) in relation to leaf toughness of host plants. *Eur. J. For. Res.* **15**, 45–52 (2012).
588. Young, J. J. A brief note on the life history of Athyma ranga (MOORE) Lepidoptera : Nymphalidae in Hong Kong <https://old.hkls.org/info-a_ranga.html> (2004).
589. Yufeng, X. *Taiwan Butterfly Illustrated Book (Part 1) Nongdie, Swallowtail, Pink Butterfly*. vol. 25. (Morning Star Publishing, 2013).
590. Yukawa, J., Nishida, R., Fukuda, H. & Inoue, R. Aristolochiaceae‐and Asteraceae‐feeding by larvae of Papilio xuthus L.(Lepidoptera: Papilionidae) in Japan: A review. *Entomol. Sci.* **22**, 355–364 (2019).
591. Yukawa, J. & Tokuda, M. *Biology of gall midges* (Springer, 2021).
592. Zacharczenko, B., Wagner, D. L. & Hatfield, M. J. A new cryptic Sympistis from eastern North America revealed by novel larval phenotype and host plant association (Lepidoptera, Noctuidae, Oncocnemidinae). *ZooKeys* **12,** 93–107 (2014).
593. Zahiri, R. *et al.* Evolutionary history of Euteliidae (Lepidoptera, Noctuoidea). *Syst. Entomol.* **48**, 445–462 (2023).
594. Zapata, A. I. & Cazaux, G. B. J. Polillas y tejidos de seda en bosques nativos de Argentina. *Revista de la Facultad de Ciencias Exactas, Físicas y Naturales* **5**, 77 (2018).
595. Zarco, A., Zapata, A. I. & Beccacece, H. M. Ciclo de vida de Psilopygida crispula (Dognin, 1905)(Lepidoptera: Saturniidae, Ceratocampinae). *SHILAP Revta. Lepid.* **43,** *321*–*329* (2015).
596. Zhang, A. H. & Li, H. H. Catalogue of Eucosmini from China (Lepidoptera: Tortricidae). *SHILAP Revta. Lepid.* ***33*,** 265–298 (2005).
597. Zhang, G.-F., Meng, X.-Z., Han, Y. & Sheng, C.-F. Chinese tortrix Cydia trasias (Lepidoptera: Olethreutidae): suppression on street-planting trees by mass trapping with sex pheromone traps. *Environ. Entomol.* **31**, 602–607 (2002).
598. Zhang, Y. Z., Hanula, J. L., & Sun, J. H. (2008). Survey for potential insect biological control agents of Ligustrum sinense (Scrophulariales: Oleaceae) in China. *Florida Entomologist*, *91*(3), 372–382.
599. Zhen, H. & Li, H. A review of Pseudohypatopa Sinev (Lepidoptera: Coleophoridae: Blastobasinae: Holcocerini), with descriptions of two new species. *Entomol. Fenn.* **19**, 241–247 (2008).
600. Zolotuhin, V. v, & Witt, T. J. A Revision of the Genus Pyrosis Oberthür, 1880 (= Bhima Moore, 1888)(Lepidoptera, Lasiocampidae). *Nachrichten Des Entomologischen Vereins Apollo* **19,** 1–31 (2007).
601. Zolotuhin, V. v. & Zahiri, R. The Lasiocampidae of Iran (Lepidoptera). *Zootaxa*, **1791,** 1–52 (2008).
