# Supplementary material for: InsectGUILD: feeding guilds of lepidopteran and hymenopteran larvae consuming Northern Hemisphere woody plants
Source: Sci Data. 2025 May 28;12:887. doi: 10.1038/s41597-025-05229-9 (PMC12119911; doi:10.1038/s41597-025-05229-9)
Supplement: Supplementary file 2 — readme_InsectGUILD [file 41597_2025_5229_MOESM2_ESM.docx]

**I nsectGUILD: feeding guilds of lepidopteran and hymenopteran larvae consuming Northern Hemisphere woody plants**

This repository contains InsectGUILD: feeding guilds of lepidopteran and hymenopteran larvae consuming Northern Hemisphere woody plants.

The complete dataset consists of 7 files; (1) “Feeding_ecology_traits.csv” data file, which contains the raw traits information table; (2) “Feeding_axes.csv” data file, which contains the coordinates obtained from NMDS analysis for each insect species. This allows to quantitatively and continuously sort species based on their feeding guild(s); (3) “Guild_membership.csv” data file, which presents, for each insect species, the maximal cluster membership degree values derived from the fuzzy clustering analysis; (4) “Insect_host_associations” data file, which presents a list of the insect species that make up the dataset by plant host(s) (n = 645); (5) “Recoded_trait_data.csv” data file, which contains re-coded raw trait information used to define the main axes of variation in feeding guilds through a non-metric multidimensional scaling (NMDS); (6) a text file “References.docx” that contains all the references included as numbers in the raw trait information table; and (7) “Analysis_code.R” file, that contains the code to reproduce the analyses.

**Table 1.** Files in the repository.

| **File Name** | **Format** | **Size** |
| --- | --- | --- |
| Feeding_ecology_traits.csv | csv | 661.0 KB |
| Feeding_axes.csv | csv | 303.8 KB |
| Guild_membership.csv | csv | 391.6 KB |
| Insect_host_associations.csv | csv | 1.04 MB |
| Recoded_trait_data.csv | csv | 249.0 KB |
| References.docx | docx | 107.0 KB |
| Analysis_code.R | .r | 13.4 KB |

**DESCRIPTION OF THE DATA FILES**

**Feeding_ecology_traits.csv**

This file contains the raw traits information table.

**Table 2.** Descriptions of each field in the Feeding_ecology_traits.csv data file.

| **Header** | **Explanation** |
| --- | --- |
| scientificName | The full scientific name of the taxon |
| order | The full scientific name of the order in which the taxon is classified. |
| Family | The full scientific name of the family in which the taxon is classified. |
| taxonID | Globally unique identifier for taxonomic names. |
| feeding_guild | Insect functional groups based on combining the information of the plant part consumed by the larvae with the information of how these are consumed.  Bud borer: species that bore into and feeds within leaf buds (i.e. undeveloped or embryonic shoot which occurs in the axil of a leaf or at the tip of a stem).  Bud chewer: species that feed externally on leaf buds.  Leaf chewer: species that lives and feeds externally on the leaves or needles and remove leaf tissue that may or may not include leaf veins.  Leaf galler: Species that feed internally within leaves, where specific metabolic interactions result in differentiation of the plant tissue and subsequent abnormal growths referred to as galls.  Leaf miner: organism that lives and feeds inside the blade of a leaf or needle, between the epidermal layers.  Shoot feeder: species that feed externally on expanding leaves/needles of the shoots.  Conospermatophage: species that feeds internally on gymnosperms cones consuming seed-bearing structures and/or seeds.  Flower borer: Species that bore into and feeds internally within flower buds, flowers/catkins or developing seeds.  Flower feeder: species that feeds externally on flowers, flower buds, and/or developing seeds.  Fruit feeder: species that feeds internally within angiosperms fruits, consuming both seed-bearing tissues and seeds.  Bark borer: species that bore into the woody portions of plants, feeding on the living tissue and disrupting transport.  Root borer: species that bore into and feeds within internal tissues of the roots.  Root feeder: species that feed externally on the roots.  Shoot borer: species that lives and feeds within expanding shoots of host plants. Shoot: the current new growth of a branch tip. |
| feeding_guild_abbreviation | Abbreviation established for each feeding guild  Ba: bark borer  Bu: bud borer  Buc: bud chewer  Cs: Conospermatophage  Fb: flower borer  Fl: flower feeder  Fr: fruit feeder  Lc: leaf chewer  Lg: leaf galler  Lm: leaf miner  Rb: root borer  Rf: root feeder  Sb: shoot borer.  Sf: shoot feeder. |
| feeding_niche | The biomass pools or transport tissues exploited by the larvae.  Flowers: the sexual reproductive structure of angiosperms, typically consisting of an axis perianth part, androecium and gynoecium.  Flower buds: primary meristems inside protective covering containing embryonic and unexpanded flower parts.  Fruits/seeds: the seed-bearing unit of angiosperms; it is the mature, ripened ovary and all of its associated protective covers, appendages, and supporting structures.  Leaves: the photosynthetic organs of a plant.  Leaf buds: primary meristems inside protective covering containing unexpanded or undeveloped leaves with axillary growing points.  Leaf gall: growth or swelling within leaves caused by hypertrophy and/or hyperplasy of plant cells, induced by an organism, which provides nutrients and shelter for that organism.  Shoots: any young, tender, succulent, current-year, aerial outgrowth from a plant.  Roots: part of the underground axial system of a plant which does not bear leaves and tends to go downwards or laterally in the soil. |
| feeding_mode | The larval feeding mode.  External (exophytic): insect feeding on plant tissues that occurs externally on the plant.  Internal (endophytic): insect feeding on plant tissues that occurs within tissue of a living plant. |
| hostplant_specialization | The diet breadth.  Monophagous: herbivore species restricted to feed on one or several plant species within a single genus.  Oligophagous: herbivore species that feeds two or more genera within one plant family.  Polyphagous: herbivore species that feeds as larva on plants from two or more families. |
| voltinism | The number of generations per year.  Univoltine: species having one generation per year.  Bivoltine: species having two annual broods.  Trivoltine: species having three annual broods.  Polyvoltine: species having two or more generations per year (number of which may be unknown or unspecified).  2-year: a species that spends two years of its life cycle in the larval stage.  3-year: a species that spends three years of its life cycle in the larval stage. |
| references | Reference identification number associated to the “References.docx” file of the species account. |

**Feeding_axes.csv**

This file contains the coordinates obtained from Non-metric Multidimensional Scaling (NMDS) analysis for each insect species.

**Table 3.** Descriptions of each field in the Feeding_axes.csv data file.

| **Header** | **Explanation** |
| --- | --- |
| scientificName | The full scientific name of the taxon. |
| NMDS1 | The first NMDS axis score of the species account. |
| NMDS2 | The second NMDS axis score of the species account. |

**Guild_membership.csv**

This file presents, for each insect species, the maximal cluster membership degree values derived from the fuzzy clustering analysis.

**Table 4.** Descriptions of each field in the Guild_membership.csv data file.

| **Header** | **Explanation** |
| --- | --- |
| scientificName | The full scientific name of the taxon. |
| Cluster.1 | The membership value associated to cluster 1 of the species account. |
| Cluster.2 | The membership value associated to cluster 2 of the species account. |
| Cluster.3 | The membership value associated to cluster 3 of the species account. |

**Insect_host_associations.csv**

This file presents a list of the insect species that make up the dataset by plant host(s) (n = 645).

**Table 5.** Descriptions of each field in the Insect_host_associations.csv data file.

| **Header** | **Explanation** |
| --- | --- |
| plant_scientificName | The full scientific name of the host plant for the lepidopteran and/or hymenopteran larvae. |
| plant_family | The family of the host plant species account. |
| plant_growth_form | Growth habit of the host plant account. |
| insect_scientificName | The full scientific name of the lepidopteran or hymenopteran larvae associated to each woody plants. |
| references | Reference identification number associated to the “References. docx” file of the species account. |

**Recoded_trait_data.csv**

This file contains the re-coded raw trait information used to define the main axes of variation in feeding guilds through a non-metric multidimensional scaling (NMDS).

**Table 6.** Descriptions of each field in the Recoded_trait_data.csv data file.

| **Header** | **Explanation** |
| --- | --- |
| scientificName | The scientific name of the species account. |
| Leaf_chewer | The membership value to the “leaf chewer” feeding guild of the species account (going from 0 to 1). |
| Leaf_miner | The membership value to the “leaf miner” feeding guild of the species account (going from 0 to 1). |
| Bud_chewer | The membership value to the “bud chewer” feeding guild of the species account (going from 0 to 1). |
| Bud_borer | The membership value to the “bud borer” feeding guild of the species account (going from 0 to 1). |
| Shoot_feeder | The membership value to the “shoot feeder” feeding guild of the species account (going from 0 to 1). |
| Shoot_borer | The membership value to the “shoot borer” feeding guild of the species account (going from 0 to 1). |
| Root_borer | The membership value to the “root borer” feeding guild of the species account (going from 0 to 1). |
| Root_feeder | The membership value to the “root feeder feeding guild of the species account (going from 0 to 1). |
| Flower_feeder | The membership value to the “flower feeder” feeding guild of the species account (going from 0 to 1). |
| Flower_borer | The membership value to the “flower borer” feeding guild of the species account (going from 0 to 1). |
| Fruit_feeder | The membership value to the “fruit feeder” feeding guild of the species account (going from 0 to 1). |
| Conospermatophage | The membership value to the “conospermatophage” feeding guild of the species account (going from 0 to 1). |
| Leaf_galler | The membership value to the “leaf ” feeding guild of the species account (going from 0 to 1). |
